# A psychometric approach to decision-making thresholds across legal and societal domains

**DOI:** 10.1093/pnasnexus/pgae592

**Published:** 2025-01-02

**Authors:** Lauren Hartsough, Matthew Ginther, Edward K Cheng, René Marois

**Affiliations:** Department of Psychology, Vanderbilt University, Nashville, TN 37240, USA; Department of Psychology, Vanderbilt University, Nashville, TN 37240, USA; Vanderbilt Law School, Vanderbilt University, Nashville, TN 37203, USA; Vanderbilt Law School, Vanderbilt University, Nashville, TN 37203, USA; Department of Psychology, Vanderbilt University, Nashville, TN 37240, USA

**Keywords:** decision thresholds, legal burdens of proof, psychometric analyses

## Abstract

What constitutes enough evidence to make a decision? While this is an important question across multiple domains, it takes on special importance in the US legal system, where jurors and judges are instructed to apply specific burdens of proof to render life-changing decisions. Civil trials use a preponderance of evidence (PoE) threshold to establish liability, while criminal trials require proof beyond a reasonable doubt (BaRD) to convict. It is still unclear, however, how laypeople interpret and apply these decision thresholds and how these standards compare to people’s intuitive belief (IB) of what constitutes enough evidence. Further, the extent to which their correct interpretation is context-dependent is currently unknown: are they unique to the legal context, or do they generalize to other contexts (e.g. medical, scientific, and perceptual) that also critically rely on decision thresholds? To compare burdens of proof across contexts requires a common parameter space. Here, we applied quantitative, psychometric analyses developed in psychophysics to compare decision thresholds across legal, nonlegal, and perceptual domains. We found a consistent pattern across domains in which BaRD was interpreted more stringently than PoE but, surprisingly, with PoE being more stringent than people’s IB. Decision thresholds were higher for legal contexts even when the costs of decision outcomes were equated. These results highlight how decisions are rendered inherently more stringently in the legal domain and suggest that laypeople’s IB are more lenient than either legal standard. These findings also illustrate the power of applying psychometrics to elucidate complex decision processes.

**Significance Statement**

What constitutes enough evidence to make a decision? While this is an important question across societal domains, it takes on special importance in the legal system, where jurors and judges are instructed to apply specific burdens of proof—namely preponderance of evidence and beyond a reasonable doubt—to render life-changing decisions. It is still unclear, however, how individuals interpret and apply these decision standards and how they compare with people’s intuitive belief (IB) of what constitutes enough evidence, not only in the legal domain but in other domains as well. Here, we found that both burdens of proof differ from individuals’ IB and that they are applied more stringently under hypothetical legal scenarios than under any other domains.

## Introduction

Decision-making has been the focus of intense academic interest, partly owing to the fact that it is a ubiquitous cognitive process, and partly because of its high-stakes implications in such varied fields as medicine, science, and the law. A salient question in these areas is what constitutes enough evidence to make a choice between different options. The US legal system, which is based on an adversarial structure in which judges and juries hear competing versions of the evidence, provides a compelling real-world context highlighting the importance of decision thresholds, as jurors are instructed to apply prescribed legal standards (i.e. burdens of proof) rather than relying on their own intuition and experience for what constitutes the proper threshold for rendering a decision. There are two main burdens of proof adopted by the US legal system, depending on whether the case is civil or criminal. In civil cases, wherein plaintiffs normally only seek compensation for damages, the standard applicable burden of proof is a preponderance of the evidence (PoE). In criminal cases, in contrast, the US Constitution demands proof beyond a reasonable doubt (BaRD), which seeks to minimize false positives (false convictions) at the cost of more false negatives, i.e. the nonconviction of a guilty offender (1). Legal scholars consider PoE to reflect the point at which the scales are tipped to one side or the other (i.e. just over the 50–50 line (2, 3)). BaRD, on the other hand, has less of a precise definition but is typically aligned with a quantified decision threshold of 90% (4, 5). This threshold loosely derives from the famed English jurist William Blackstone’s statement that “it is better that 10 guilty persons escape than that 1 innocent suffer” (5, 6).

There is a strong impetus for ensuring that these burdens of proof are fairly and consistently applied given the prevalence and high cost of such decisions for both the parties to a case and society more generally (7–12). Thankfully, studies of judges suggest that their application of the burdens of proof generally conforms to jurisprudence: most judges place PoE at 50–55% (2, 3); but see (13), and their estimates for BaRD are also in accord with the normative expectations of close to 90% (2, 3). Most civil and criminal cases that make it to trial, however, are not decided by judges but by a jury of laypeople who have little to no legal expertise in the law and its application. What is more, there is considerable evidence that their applications of the burdens of proof do not conform to the justice system’s expectations.

Jurors will often receive instructions regarding the appropriate burden of proof, but these instructions are almost always purposefully vague. And while it is ubiquitous for legal scholars and judges to describe (and even teach) the legal standards in terms of probabilities, in most jurisdictions judges and attorneys are specifically prohibited from instructing jurors using the same quantitative framework they use themselves (14). Instead, jurors are often instructed to interpret them as they will. It is therefore not surprising that studies assessing jurors’ interpretations of burden of proof instructions showed that these do not align well with the normative thresholds and judges’ interpretation of the thresholds. With respect to BaRD, laypeople’s estimates range between 0.65 and 0.90, with most falling well below the expected threshold (3, 15–17). In contrast, potential jurors’ estimates of PoE were around 75% (Simon and Mahan (3)), indicating that their application of this civil burden of proof is far more conservative than judges’ application and what the law prescribes. It is conceivable that these discrepancies between judges and jurors are due to jurors favoring their own decision thresholds over the provided standards. Consistent with this possibility, individuals who convict tend to ascribe lower estimates of BaRD compared with those who acquit, indicating that people may adjust interpretations of the burdens of proof to align with their own decision thresholds (18, 19).

Given that jurors’ estimates for BaRD are typically less stringent than both judges’ and the normative standard (2, 3, 15, 16), while estimates for PoE are more stringent than the intended construct (3), it has been suggested that laypersons possess a single intuitive decision threshold falling between BaRD and PoE and this threshold serves as an anchor across burdens of proof (2, 3, 15, 16). Given all these issues with the legal standards, it is surprising that no studies have yet sought to systematically determine how they compare to individuals’ intuitive decision thresholds. Put another way, it is difficult to gauge the extent to which jurors are being influenced by their own intuitions of what constitutes enough evidence because we have little knowledge of what those intuitions correspond to.

Equally unexplored is how the application of these legal burdens of proof compares to decisions in domains other than a legal one. Are these decision standards interpreted in a particular way because of the legal context they are normally applied in, or do they generalize across contexts? To take PoE’s core definition as an example is the “tip of the balance” threshold interpreted similarly regardless of whether it is applied in law, medicine, science, or even perception? Or does it have a specific meaning—and application—in the legal domain? The finding that estimates of legal burdens of proof are markedly different between judges and jurors suggests that they are context-dependent, in this particular case, based on legal expertise. Are these contextual effects magnified if the decision standards are considered outside of the legal domain? Answering such questions will help define the contextual space in which legal burdens of proof are interpreted and applied.

Comparing the effects of different burdens of proof instructions across multiple domains requires bringing them into a common experimental space. Here, we adopt a psychometric approach widely applied in psychophysics. Psychometric functions are simple mathematical functions that express how changes in a given variable/parameter contribute to changes in decisions (20). This analytical approach has not been widely applied to the understanding of decision-making in such a complex domain as the law (though see (21) for a cogent application of signal-detection theory to forensic pattern matching and (22) for the novel application of a high-throughput, web-based approach to investigate evidence-based decision-making in criminal scenarios). Yet, a psychometric analysis is advantageous due to its simplicity and ability to characterize the relationship between dependent and independent variables with just four parameters: one defining the point (threshold) at which the stimulus strength along the abscissa is sufficient to change the decision (e.g. from no to yes, customarily defined as the 50% mark), one defining the strength of the relationship between the variable and the decision (i.e. its slope), and one each defining the upper and lower asymptotes that convey the likelihood of making a certain decision when the stimulus strength is at its maximum and minimum values, respectively (23, 24).

The advantages of applying a psychometric approach to both legal and nonlegal forms of decision-making are multifold: it not only provides a quantitative and sensitive assessment of the effects of manipulating variables on decision-making, it also can reveal specific effects that these variables may have on distinct parameters of the psychometric function, thus potentially uncovering not just whether but also how different variables affect decision-making. Furthermore, while previous studies of legal decision-making typically manipulated evidence strength dichotomously as either weak or strong (16, 18, 25), a psychometric approach lends itself to examining decisions across a range of evidence strengths, including moderate, ambiguous strengths. This is particularly applicable to legal decision-making as cases that are most likely to go to a jury trial where burdens of proof are applied by laypeople are those with equivocal evidence (26, 27).

Here, we implement a psychometric approach to assess and compare decision thresholds across legal, nonlegal, and psychophysical domains. The main objectives are to contrast the effects of the civil (PoE) and criminal (BaRD) burdens of proof on decisions relative to subjects’ intuitive beliefs (IB, no instruction) and to determine the extent to which the effects of these decision standards are contingent on specific contexts.

## Effects of burdens of proof across legal, nonlegal, and psychophysical domains

The first two experiments assessed the effect of legal burden of proof instructions on decisions across both legal and nonlegal textual (e.g. medical, scientific, financial) and perceptual contexts. In experiment 1, 5,468 online participants made a single binary decision to a brief textual scenario that varied, between subjects, the scenario context (legal or nonlegal), the strength of the available evidence (from 20 to 100% probability), and the decision criteria instruction (IB, PoE, or BaRD). For example, a participant that was randomly assigned to the BaRD instruction with a legal scenario and an 80% probability would first read a one-paragraph-long scenario that would describe a criminal act that may have been perpetrated by the protagonist, including the 80% probability with which investigators were able to link the available evidence to the protagonist. They then read the decision criteria definition for BaRD and subsequently made a decision as to whether or not they believed the protagonist committed the crime according to the decision criterion (see Supplementary Section S1).

Experiment 2 sought to assess the effect of the same burdens of proof in the perceptual context. We used a dot motion coherence task that has been used extensively to study decision processes using psychometric functions (28–30). As such, experiment 2 represents an extreme test of the contextual dependence of burdens of proof by evidence strength, as the characteristics of the evidence to be considered are clearly distinct between the legal and perceptual domains. In the dot motion coherence task, participants decided whether they believed there was coherent directional motion among dots moving about on a computer screen (see Supplementary Section S4). For any given trial, the evidence strength, corresponding to the percent of the dots that moved coherently in a particular direction, was varied between 0 and 100%, with each participant assigned to one particular instruction type (IB, PoE, or BaRD). Note that the design of experiment 2 departed notably from the online single-trial, scenario-based experimental designs used in experiment 1 in that each of the 72 participants performed 180 trials in the laboratory as a necessary compromise for carrying out a perceptual task that typically involves the acquisition of hundreds of trials per subject to derive the appropriate signal-to-noise ratio, though we ascertained in an online version of this experiment (see Supplementary Section S5) that the results presented below are qualitatively the same when a single dot motion trial is presented to over a thousand participants.

**Fig. 1.**
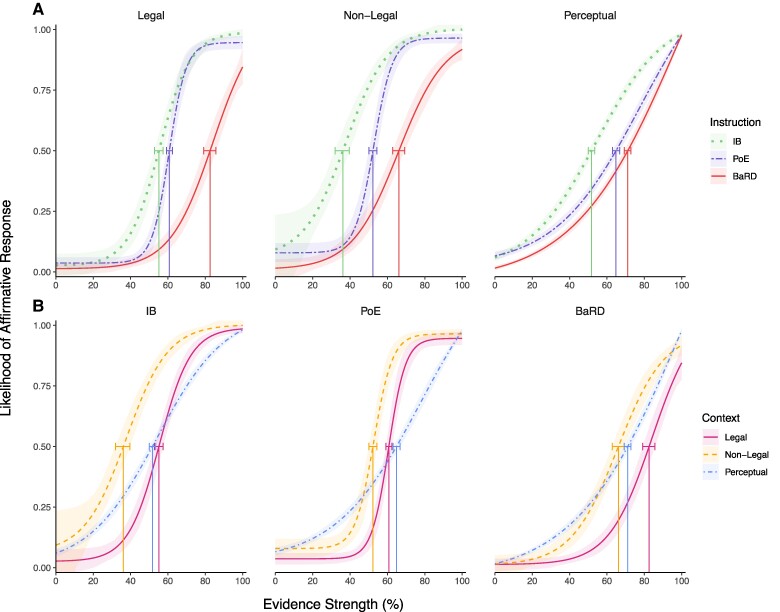
Likelihood of an affirmative response by evidence strength (participants’ subjective evidence strength for legal and nonlegal, % dot coherence for psychophysical). Shaded regions are 95% CIs estimated via 1,000 bootstrap samples. Decision thresholds are marked with vertical lines and 95% error bars. A) The effect of instruction within each context and B) the effect of context within each instruction.

Figure 1A shows the effect of instruction (BaRD, IB, and PoE) within each context, while Fig. 1B presents the effect of context (legal and nonlegal from experiment 1, perceptual from experiment 2) within each instruction. The plots are the psychometric functions of the likelihood of an affirmative response by evidence strength (i.e. participants’ subjective estimation of evidence probability for the scenario-based legal and nonlegal contexts and % dot motion coherence for the perceptual context).

**Fig. 2.**
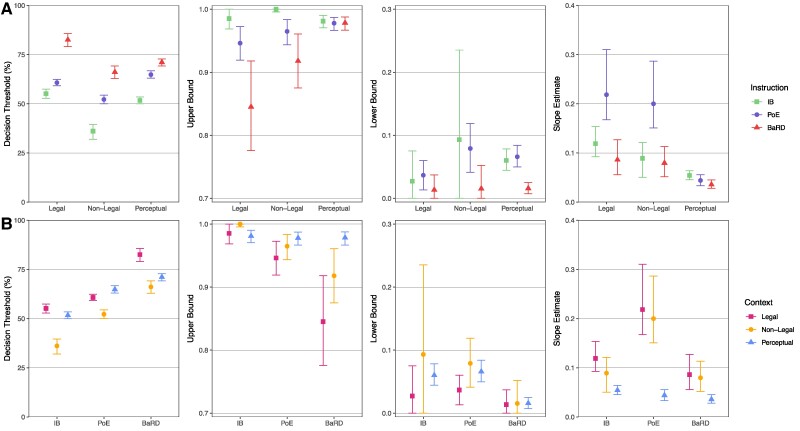
Decision thresholds (% evidence strength at likelihood of affirmative = 0.50), upper bounds (likelihood of affirmative at % evidence strength = 100), lower bounds (likelihood of affirmative at % evidence strength = 0), and slope estimates (delta likelihood/delta % evidence strength at likelihood of affirmative = 0.50) by instruction and context. 95% CIs estimated via 1,000 bootstrap samples. Top row A) shows the effect of Instruction within each context, while bottom row B) shows the effect of context within each instruction.

For all three contexts, there was an effect of decision criteria instructions on participants’ decisions such that they were most conservative in the BaRD condition, followed by PoE and then IB (Fig. 2; IB vs. BaRD: −26.14 [−29.08, −22.65]; IB vs. PoE: −9.08 [−11.77, −6.52]; PoE vs. BaRD: −17.06 [−19.55, −14.10]). This was further borne out in statistical comparisons of the decision thresholds within each context (see Tables S1.5 and S4.2 for all pairwise comparisons), with higher thresholds indicating that participants required stronger evidence to make an affirmative response. The BaRD threshold was significantly more stringent than both PoE and IB, and the IB threshold was significantly more lenient than PoE. The effect of instruction was similarly reflected in the upper bounds of the legal and nonlegal contexts (Fig. 2); IB’s upper bound was greatest (least stringent), followed by PoE and BaRD, with a significant difference between IB and BaRD in both legal and nonlegal contexts and between IB and PoE in the nonlegal context, suggesting that even at higher levels of evidence strength, participants are more conservative in their decisions after receiving legal instructions. The effect of instructions on upper bound was not seen in the perceptual context, most likely because dot motion coherence was irrefutable at 100% coherence. The perceptual experiment, however, did reveal a similar effect to the legal and nonlegal contexts for the lower bound, with more liberal decisions in the IB and PoE conditions compared with BaRD (Fig. 2). In sum, these results suggest that participants’ decisions are partly consistent with the intent of the legal system in that they apply a more conservative decision criterion for BaRD than PoE. Contrary to expectations, however, participants are more conservative in response to PoE instructions relative to when they receive no legal instruction (IB). Interestingly, the slopes for the PoE instructions were steeper (i.e. higher) for the legal and nonlegal contexts than for the perceptual contexts and for the BaRD and IB instructions (Fig. 2, Tables S4.3 and S4.4), suggesting that the “tip of the balance” instruction led to a sharper delineation between affirmative and negative responses, as intended by PoE’s burden of proof. (The absence of such sharp sigmoid delineation in the perceptual task is likely a result of the low number of trials employed in the present experiment—compared with the 100 s of trials in a standard psychophysical experiment—that led to overly conservative judgments).

We also examined the effect of context on participants’ decisions and found that decisions were more stringent in the legal than in the nonlegal context overall (legal vs. nonlegal: 13.54 [11.35, 15.82]), also evidenced by significantly higher decision thresholds in the legal condition with pairwise comparisons (Table S1.5). When compared with the perceptual context, the higher stringency of the legal context only applied for the BaRD instruction (Table S4.3), again likely due to the conservative responses for PoE in the perceptual context (see above). The nonlegal context had the least stringent decisions across instruction types (Tables S1.5 and S4.4). With respect to the slope estimate, the only marked difference was the shallower slopes in the perceptual context, again owing to a general conservative shift in decision criterion for this context relative to the two other contexts (Tables S4.3 and S4.4). No systematic effect of context was found for the upper and lower bounds (but see above for the differential effect of context for the BaRD instructions).

Finally, we compared the decision thresholds for each condition (instruction × context) to the prescribed (normative) legal standards for PoE (just over 50%) and BaRD (90%) by determining whether these standards fell within the 95% CI for each threshold estimate (Fig. 2, Tables S1.4 and S4.1). Thresholds for BaRD were significantly lower than the 90% standard in all contexts (legal: 82.55, 95% CI [79.09, 85.68]; nonlegal: 66.12, 95% CI [62.84, 69.19]; perceptual: 71.11, 95% CI [69.24, 72.81]). The PoE threshold was significantly >50% in the legal and perceptual context (legal: 60.74, 95% CI [59.21, 62.40]; perceptual: 64.83, 95% CI [62.99, 66.78]), while the interval for the nonlegal context was just over 50% (52.21, 95% CI [50.05, 54.40]), consistent with the prescribed standard. The IB threshold was significantly >50% for the legal context (55.14, 95% CI [52.81, 57.42]), significantly <50% for the nonlegal context (36.12, 95% CI [31.95, 39.58]), and not significantly different from just above 50% for the perceptual context (51.69, 95% CI [50.01, 53.43]). Thus, both the PoE and BaRD legal decision standards fell outside the prescribed range—though in opposite directions—in the legal and perceptual contexts.

Taken together, the results suggest that the application of legal standards is generalizable across a wide range of domains, as we found similar trends using legal and nonlegal scenarios as well as a classic perceptual motion coherence task. The results also suggest that participants generally adopted a more stringent decision criterion in the legal than in the nonlegal or perceptual context. The only exception to that finding was the high decision threshold for PoE instruction in the perceptual context, possibly owing to participants interpreting PoE to mean that a preponderance of the dots had to move together, and they ostensibly perceived this to only be the case when about two-thirds of the dots did so.

### Why are legal decisions more stringent?

Our finding that decision thresholds were more stringent across all instructions in the legal vs. nonlegal context, even using the same scenario-based experimental design, may indicate that some aspect of the legal context renders decision-makers more guarded in returning an affirmative decision. We considered three different possibilities to this difference.

First, the wording of the evidence strength differed between contexts. Specifically, for nonlegal scenarios, the evidence strength related to the same event/action that the participants ultimately rendered a decision on (e.g. the evidence strength referred to the % probability that a patient’s DNA sequence was indicative of a disorder, and participants were asked to judge whether the patient had that disorder). By contrast, in the legal scenarios, the objective evidence strength referred to the level of certainty that the physical evidence associated with the wrongdoing was linked to the protagonist, but participants’ decisions focused on whether the protagonist had committed that wrongdoing (e.g. the evidence strength referred to the % probability that the DNA was Mark’s but participants were asked to judge whether Mark committed the wrongdoing). While subtle, this may have led participants in the legal context to believe that Mark did not commit the wrongdoing even though they may have believed that the evidence was linked to him, despite our attempts to phrase scenarios such that the evidence could only have come from the perpetrator. This would result in fewer “affirmative” responses in the legal context (relative to the nonlegal context) at a comparable evidence strength. To determine whether the greater stringency in the legal vs. nonlegal context can be explained by this difference in scenario language, we ran a control experiment (see Supplementary Section S2) in which we modified our legal scenarios to match the language in the nonlegal scenarios so as to directly link the evidence strength to the wrongdoing that participants rendered a judgement on. The results indicate that presenting the legal scenarios using language that matched that of the nonlegal scenarios did result in less stringent legal decisions, but not to the extent that it can fully account for differences between the legal and nonlegal contexts. This suggests that the more stringent decisions in the legal context are due, at least in part, to properties specific to the legal domain.

Another consideration applies specifically to the overly stringent application of PoE’s burden of proof in the legal context. It is possible that while our legal scenarios described acts that could be considered either criminal or civil wrongdoings, participants viewed them as more criminal than civil. Thus, the overly stringent PoE standards observed in this and the previous study (3) may be driven in part by participants inferring a criminal context. This would not apply to the nonlegal context, for which the PoE threshold was consistent with the prescribed 50% standard, presumably because there can be no implication of any such criminal context. To determine whether the overly stringent application of the PoE standard in the legal context can be explained by participants inferring a criminal context, we ran a control experiment in which participants responded to the PoE instruction for a legal scenario in an explicitly civil context (i.e. protagonist faces litigation for wrongdoing). We found that presenting the PoE instruction in an explicitly civil legal context did not result in a more lenient application of the instruction compared with the original experiment (see Supplementary Section S3). This suggests that the overly stringent application of the PoE standard is not due to participants inferring a criminal context, and provides further evidence that the more stringent decisions observed in response to legal vs. nonlegal scenarios are due to specific aspects of the legal context.

A third possibility is that the distinctive stringency of legal decisions is due in part to participants implicitly attaching a punishment to a culpability verdict despite our attempt to keep the prompt language neutral (i.e. no use of “guilty” or “responsible”), which may increase decision thresholds due to the inferred negative consequences to the defendant. It is common knowledge that more severe crimes generally call for harsher punishment, and it is reasonable to expect that individuals are aware that defendants will face consequences if found guilty. In that context, participants may be more averse to a false positive (convicting an innocent person) than a false negative (acquitting a guilty person) in the legal context; indeed, the BaRD standard is founded on this very principle, favoring false acquittals to false convictions at a 10:1 rate (4, 5). Furthermore, several of the nonlegal scenarios used in experiment 1 may have encouraged more lenient thresholds as a false positive would be preferable to a false negative (e.g. it may be better to proceed with a treatment on a patient who is incorrectly diagnosed as positive for a disease than failing to apply the treatment to a patient who is wrongfully diagnosed as negative for the disease).

In experiment 3, we tested the hypothesis that legal decisions are more stringent because they conjure inherent costs/consequences associated with such decisions. We explicitly informed participants of the potential outcomes of their decision before they rendered it, and manipulated the costs associated with these decision outcomes both within and across scenarios. We also assessed how the effect of cost is influenced by the legal instruction (PoE, BaRD, or no instructions) and by the contextual domain (i.e. legal vs. nonlegal). Thus, the experimental design of experiment 3 was similar to experiment 1 except for the inclusion of a decision cost statement following the instructions statement that stated the consequences of rendering an affirmative response (e.g. that the protagonist had committed the crime or that they were afflicted with a disease). For each scenario, the stated costs were either in dollar amounts or duration, each varying between a “low” and “high” cost level (e.g. $10,000 vs. $100,000 of legal or medical costs, or 1 vs. 10 years of incarceration or incapacitation) (see Supplementary Section S6). Finally, we lengthened the nonlegal scenarios to make them comparable in the number of words to the legal scenarios. In experiment 1, nonlegal scenarios consisted of two sentences while legal scenarios consisted of five to seven to establish the “closed” environment for the wrongdoing. Equating scenario length serves to eliminate another possibility for the differences between legal and nonlegal scenarios.

**Fig. 3.**
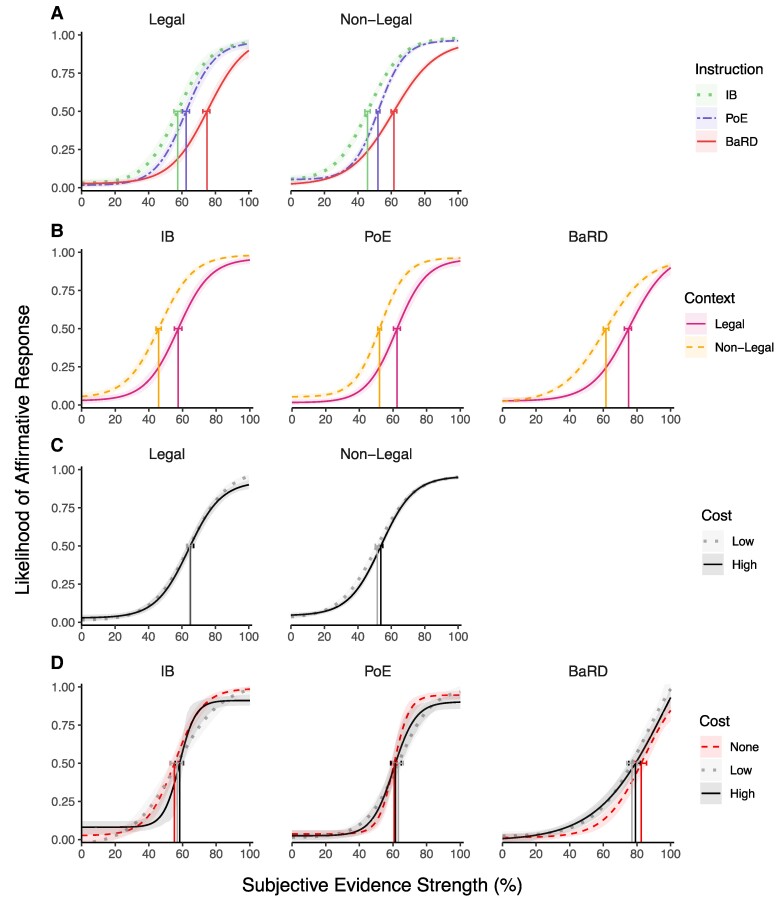
Likelihood of an affirmative response by subjective evidence strength. Shaded regions are 95% CIs estimated via 1,000 bootstrap samples. Decision thresholds are marked with vertical lines and 95% error bars. A) Effect of instruction within the legal and nonlegal contexts. B) Effect of context within instruction. C) Effect of low vs. high relative costs within context collapsed across instruction types. D) Comparison of no cost data of experiment 1 to the low- and high-cost data of experiment 3 by instruction types for the legal context. Legal scenarios are identical between experiments 1 and 3.

As in experiment 1, we collapsed the nonlegal domains into a single nonlegal context (see Supplementary Section S6B for results broken down by domain). We first compared the effect of instructions on decision thresholds within context (Figs. 3A and S6.2) and the effect of context within instruction (Figs. 3B and S6.3), collapsing across decision costs. Consistent with experiment 1, the effect of instructions on decision thresholds was similar across all contexts such that IB < PoE < BaRD (overall IB vs. BaRD: −16.90 [−18.80, −14.82], IB vs. PoE: −5.77 [−7.66, −4.01], PoE vs. BaRD: −11.13 [−12.95, −9.32]; legal vs. nonlegal: 12.84 [11.15, 14.28]), and decisions were most stringent in the legal context across all instructions with a decision threshold significantly greater than for the nonlegal contexts (see Table S6.1 for all pairwise comparisons). We did not observe clear patterns of significant differences between the slope or asymptote parameters for this or the following analyses, so we focus our results on the decision thresholds.

While the effects of context and instructions were predicted, that was not the case for the effect of costs. Specifically, we did not observe an effect of cost within any of the contexts (Fig. 3C), with no evidence of a trend between high and low costs (Fig. S6.4). The designation of a cost as low vs. high was relative to each scenario, and the low cost in one scenario could be greater than the high cost of another scenario within the same context (e.g. low-level cost for murder = 10 years in prison, high-level cost for prescription drug theft = 5 years in prison), which could complicate the interpretation of data collapsed across low and high costs. To assess the effect of cost within each context unconfounded by the relativity of the costs, we compared the absolute cost amount provided to participants in each context. This absolute cost analysis yielded the same result as the relative cost analysis: cost amount had little impact on decisions (see Figs. S6.5–S6.8 and Tables S6.2–S6.5):overall, decision thresholds were not significantly different between absolute cost amounts within the legal and nonlegal contexts.

While varying the costs had no effect on decision thresholds, it is possible that adding any cost to a decision that would otherwise have no cost could affect decision thresholds. To determine whether including any cost (low or high) influenced decisions, we compared decision parameters obtained in the legal context for experiment 3 (which had either low or high decision outcome costs) to those from experiment 1 that included identical legal scenarios but without any decision outcome cost given. Here again, save for a small difference between the high cost and no cost for the IB instruction, we did not observe differences between the costly decision conditions (either low or high) and the no-cost condition (Fig. 3D, Table S6.6). Thus, there were no differences in participants’ decision thresholds regardless of whether there were no costs or varying amounts of costs to the decisions.

**Fig. 4.**
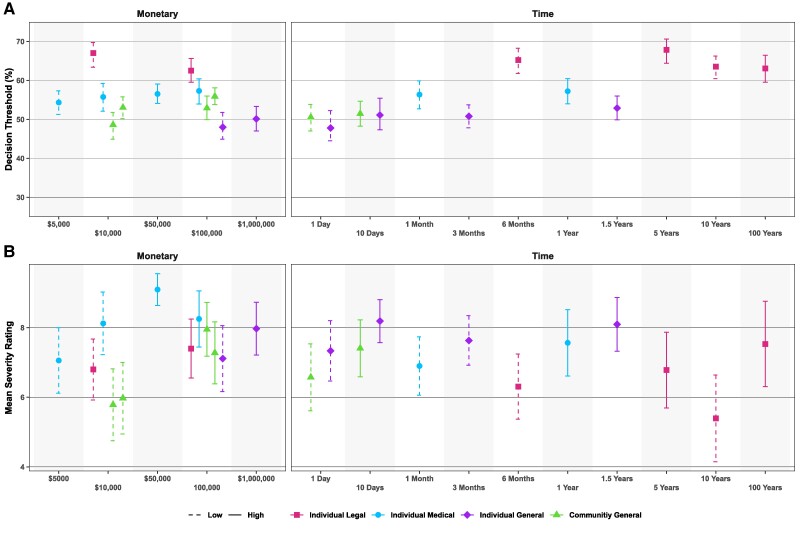
A) Decision thresholds with 95% CIs by cost amount in ascending order. B) The mean severity ratings ± 95% CIs by cost amount in ascending order. The left panels include monetary costs, while the right panels include duration costs. Color and shape of data points designate the scenario context. Low costs are represented by dashed lines, while high costs are represented by solid lines (low vs. high costs are relative terms to refer to differential costs applied within each context; see Supplementary Section S6A).

The distinctive effects of societal contexts and decision costs on decision thresholds are best revealed when plotting decision thresholds by ascending cost amounts across contexts (Fig. 4A). The data are not collapsed across nonlegal domains here so that we can compare the effects of absolute monetary and time costs. First, this figure reveals how little the decision thresholds change with ascending monetary or time costs, consistent with the results described above. Instead, we observed the same trend as in Fig. 3B, with the legal decisions being more stringent than any of the nonlegal decisions. The predominance of the effect of contexts over the effect of costs is most striking when comparing decision thresholds across contexts for specific duration costs or financial costs. For example, the 6-month legal cost has a higher decision threshold than medical costs that are twice as long (Fig. 4A). This may be explained, however, by the fact that participants deem 6 months of incarceration as being far costlier than 1 year on a liquid diet. Comparison across domains is more appropriate when the monetary costs are comparable, which is the case for the $10,000 and $100,000 monetary costs. Pairwise comparisons within the $10,000 cost amount found that the legal threshold was significantly greater than all of the nonlegal thresholds, while the threshold for the medical domain was significantly greater than for individual general insurance (Table S6.7). Similar patterns were obtained for the $100,000 cost (Table S6.8). Most strikingly, the decision thresholds for the $10,000 legal costs were even greater than the decision thresholds for all the $100,000 nonlegal costs. Thus, decision thresholds in the legal domain are more stringent than in other domains not only when decision costs are explicitly equated across contexts, but even when the financial costs are higher in the other contexts.

It could be argued that we did not observe an effect of costs because the low and high costs were not sufficiently different from each other for the participants to code them as distinct. For that matter, these costs may not be sufficiently different from no costs at all to affect decision thresholds. We explore this possibility in a separate experiment by having participants rate on a Likert scale the severity of the different scenario costs used in experiment 3 (see Supplementary Section S7). Participants clearly distinguished between the severity of the low and high costs (Fig. 4B). Some of the nonlegal costs were even rated as more severe than the individual legal costs, yet the legal context had the most stringent decision thresholds. These results rule out a failure to discriminate between decision costs as a cause for the lack of an effect of cost outcomes on decision thresholds in experiment 3. Overall, the cost experiment suggests that the perceived severity of the cost associated with a decision does not influence the decision threshold and that legal decisions are applied more stringently than other types of decisions.

## Discussion

In a series of experiments, we implemented a psychometric approach to quantitatively assess the effect of legal standards of proof (PoE, BaRD) on decision thresholds to compare these burdens of proof to people’s own IB in both legal and nonlegal contexts. There are three main takeaways from these experiments: (ⅰ) our intuitive decision standards tend to be more liberal than not only the BaRD standard but also PoE across contexts, (ⅱ) legal decision standards are more stringent than nonlegal judgments across widely different contexts (i.e. medical, scientific, and perceptual), and (ⅲ) this higher stringency is not due to the potential costs associated with rendering a legal (or nonlegal) decision. Moreover, the present study reveals the analytical power of the psychometric approach in investigating complex decision standards within and across domains.

An important aim of the present study consisted in assessing how decision parameters for the BaRD and PoE instructions compared not only to each other but especially to participants’ decisions in the absence of any legal instruction (i.e. IB). Across both legal and nonlegal contexts, including the perceptual domain, decision thresholds (at 50%) were highest for the BaRD instruction, followed by the PoE instruction and lastly by the IB condition. That the BaRD thresholds were more stringent than the PoE thresholds is consistent with the ideal distinction put forth by the legal system (1, 4) as well as with previous work quantifying these burdens (3). This appears to be a robust trend as it held true across methodologies and contexts in the present study. Another finding consistent with prior work (2, 3, 15, 16) is that the decision threshold estimates were more stringent than legally prescribed for PoE and more liberal than prescribed for BaRD. PoE is meant to fall around a “tipping of the scales” at just above 50%. However, previous studies have found that jurors and judges alike tend to interpret PoE as having a threshold >50%, with estimates for judges ranging between 50 and 55% and jurors estimating PoE as high as 75% (2, 3). Our findings suggest that individuals are overly stringent in applying the PoE threshold not only in the legal domain but even in the absence of a legal context (e.g. under nonlegal scenarios and perceptual contexts). By contrast, we found that decision thresholds for the BaRD instruction were more lenient than the prescribed 90% threshold, consistent with previous studies (2, 3). Our experiments did not have particularly high stakes; however, it may be the case that actual jurors would apply a more stringent BaRD threshold when dealing with a real legal case with actual consequences. As a counter to this argument, the PoE interpretations were overly stringent despite the lack of real-life consequences so it seems unlikely that this alone accounts for the discrepancy. One notable exception to the overly lenient application of the BaRD standard was the participants’ conservative response to this instruction when the evidence was seemingly irrefutable (i.e. 100%), regardless of scenarios (legal or nonlegal) or domains (cognitive or perceptual) tested. This finding suggests that people may have an inherent aversion to accepting absolute levels of proof.

The overly stringent application of PoE and overly lenient application of BaRD observed in this and previous studies (2, 3, 15, 16) has been interpreted as resulting from individuals shifting each standard to align more closely with an intuitive decision threshold that falls somewhere between them. The finding that participants’ IB thresholds were more lenient than the PoE thresholds is inconsistent with this interpretation. Indeed, participants’ IB thresholds fell closer to the 50% normative standard than PoE thresholds for both the legal and nonlegal contexts, which suggests that intuitive decision thresholds may be quite liberal and naturally reflect the idea of “more likely true than not” more faithfully than the intended PoE instructions. We surmise that pattern jury instructions adopted by federal courts for PoE may be over-specified, adding an unintended gravitas to the instructions that paradoxically brings decision thresholds further away from the near 50% criterion desired by the law and espoused by people’s IB. It should be noted, however, that our instructions did not adhere to the legal terms typically prescribed for applying the PoE and BaRD standards—namely “liable” or “responsible” for the former and “guilty” for the latter—in order to have a common linguistic ground across all contexts (it would be nonsensical to use “guilty” in the medical context, for instance). It is possible that the inclusion of these legal terms could differentially impact decision thresholds across instructions. Clearly, our present findings call for further studies in that respect.

A robust finding of the present study is the systematic effect of context on decision-making, as decision thresholds were consistently greater for the legal compared with the nonlegal context across instruction types. We assessed several possibilities to account for those results. We first ruled out that this effect may be due to subtle differences in scenario phrasing (see Supplementary Section S2), inferring a criminal context for PoE (see Supplemental Section S3), or even just differences in scenario length and complexity. Yet another experiment directly addressed whether these findings could be due to the implicit costs that participants inferred while making legal decisions. The main upshot of experiment 3 is that laypeople’s decisions were unaffected by the explicit costs manipulation despite considerable differences between low and high costs, which differed as much as 10-fold. Thus, like Freedman et al. (31) and Teitcher and Scurich (17), we did not observe an effect of punishment severity on legal decisions. Even a wrongdoing associated with 6 months of incarceration led to the same decision threshold than a far more serious wrongdoing tied to a 100-year-long incarceration. The fact that participants were not exposed to the two levels of decision costs for the same scenario was likely important in the outcome of our results as it prevented reference effects in punishment costs that plagued earlier studies (see (17)). Importantly, however, experiment 3 still showed that legal decisions were inherently more conservative than any other contexts. It is striking that we found that the context in which the decision is rendered is far more important in determining the decision threshold than is the cost of making that decision. This is most obvious in the comparison of monetary costs because we presented the same cost amounts in multiple contexts ($10,000 or $100,000) and found that even for the same cost to an individual, the legal context still resulted in more stringent decision thresholds. Furthermore, this effect was so pronounced that a $10,000 punitive cost to an individual in the legal context led to more stringent decisions than $50,000, $100,000, and even $1,000,000 costs to individuals in other contexts. We suggest that laypeople may be more reluctant to render an affirmative legal judgment not because they anticipate specific monetary or imprisonment consequences to the defendant, but rather because it conjures an inherently detrimental impact that such a judgment will have on the life of the defendant. In other words, the legal domain may implicitly call for more stringent decision criteria simply because convictions are inherently associated with harmful consequences to the defendant’s reputation, liberty, and/or financial prospects. In contrast, decision-making in other contexts is not inherently or necessarily detrimental. For example, in the medical domain, the cost of the treatment, whether in dollars or in time, may ultimately lead to improved long-term health outcomes for the patient. Indeed, the legal context may be unique in that substantial costs (monetary, incarceration, and even capital punishment) can be imposed on an individual without there being any potential benefit to the individual themselves (though some may argue for the correctional value of incarceration). In that context, laypeople may be more stringent in rendering legal decisions because they are hesitant to impose a punitive cost on a person that may be innocent after all (as suggested by subjective evidence probabilities), whereas they are less stringent in the other contexts because they infer redeeming values to these costs (e.g. a person’s improving health). In some ways, the findings that legal decision costs appear to have little impact on the decision criterion and that legal decisions are rendered more conservatively than decisions in other contexts regardless of the actual punitive costs may be welcome news to the legal system. Decisions about guilt should not, ideally, be influenced by the outcome of that decision—the criminal justice system separates the trial phase and the sentencing phase, after all—and legal decisions should be inherently conservative given the serious implications they have upon the life of the individual.

Much of the robustness of our findings can be ascribed to the use of a psychometric approach as it provided a common analytical framework to compare and quantify the application of burdens of proof across legal, nonlegal, and even perceptual domains. Observing the same effect of instructions (i.e. IB < PoE < BaRD) across such different contexts indicates that this finding is not simply due to methodological considerations such as differences in scenario content or providing a frequentist probability estimate. Moreover, each of the four psychometric parameters reveals distinct features of decision-making beyond what can be glanced at by just looking at single measures of decisions. For one, the decision threshold provides an estimate of the evidence strength needed to make a “yes” response more likely than a “no” response (i.e. evidence strength where *y* = 50%). These decision thresholds were useful in not only demonstrating the relative decision stringency between our different conditions, but also providing an estimate of the discrepancy between the application of the legal PoE and BaRD standards and their prescribed intent (just over 50 and 90%, respectively). By contrast, the slope provides an estimate of the strength of the relationship between the probability of the evidence and the decision outcome. The PoE instruction applied to the legal context illustrates well how these two psychometric parameters inform different aspects of the decision-making processes: while the decision threshold was significantly higher than 50% (meaning participants applied the standard more stringently than intended), the slope was steeper than for the IB and BaRD instructions (meaning subjects’ decisions were more responsive to changes in evidence under PoE than the other conditions). The latter finding is in line with the concept that the PoE standard is a tipping point between two decisional outcomes, even though we found that tipping point to be more stringent than prescribed by the judicial system. Finally, the lower and upper bounds of the psychometric function can reveal limitations in the relationship between evidence and decision, as exemplified by the diminutive upper bound of the BARD instruction in legal scenarios of experiment 1, thus revealing how BaRD instructions can lead to conservative decisions even under absolute (100% probability) conditions. Evidently, the application of the psychometric approach to legal decision-making provides a powerful tool to dissect even complex decision processes.

Despite the power of this approach, we acknowledge some potential limitations. First and foremost, our emphasis on rigorous experimental control and parametric manipulations favors scientific interpretability at the expense of external validity. For example, in real-life court cases, multiple pieces of evidence are considered together before drawing a legal judgment, and jurors hear from conflicting points of view. It would be worthwhile to determine whether our findings would be replicated in mock trial situations where a quantified error rate to some inculpatory or exculpatory testimony would be introduced by an expert. Furthermore, presenting a wider range of potential costs in mock trial situations could help determine whether there is a point at which the decision cost may have an impact on decision outcomes.

We also recognize that the present findings apply only to single individuals’ judgement. Juries in the court of law make decisions as a group and need to reach either a unanimous (in criminal trials) or near-unanimous (civil cases) decision. The burden of proof thresholds of individuals vary—as evidenced by the error bars in our data—and a consensus must develop across jurors for a verdict to be reached (absent of a hung jury). How group dynamics would affect the present findings is a fascinating topic that merits further studies. Finally, we are cognizant of the law’s reticence, if not aversion, to naked statistics and probabilities (see (32) and references therein), and rare are the cases that are swayed by clear-cut statistical evidence. It does not mean, however, that the law should not aspire to being more receptive to quantitative assessments when those bear relevance to the aims of the burdens of proof ascribed by the judicial system.

These limitations notwithstanding, the upside of comparing burdens of proof and decision standards across a wide range of domains by means of a common parameter space is substantial. Specifically, the present research applies a psychophysical approach to examining legal decision-making and comparing it with other societal domains and to people’s intuitive decision standards. By doing so, the findings from this and future work can isolate how variables of interest, such as juror instructions, prejudicial information, or punishment outcomes, can influence legal decision-making and help determine how legal decision-making is distinct from making judgments in other contexts. As such, these studies may yield insights into the foundational processes of decision-making in not only law, but also a broad range of societal domains where decisions are at once complex and consequential.

## Materials and methods

Participants in all experiments provided informed consent, and the experimental protocol was approved by the Vanderbilt University Institutional Review Board. The details of all experimental procedures and data analyses are provided in Supplementary material, Extended Methods.

The data and code for the primary analyses underlying this article are available on Open Science Framework DOI 10.17605/OSF.IO/GR97N, at https://osf.io/gr97n. This study was part of dissertation work by Hartsough (33).

## Supplementary Material

pgae592_Supplementary_Data

## References

[1] 397 US 358. 1970.

[2] McCauliff CMA . 1982. Burdens of proof: degrees of belief, quanta of evidence, or constitutional guarantees? Vanderbilt Law Rev. 35:1293.

[3] Simon RJ, Mahan L. 1971. Quantifying the burdens of proof: a view from the bench, the jury, and the classroom. Law Soc Rev. 5(3):319–330.

[4] Laudan L . 2003. Is reasonable doubt reasonable? Legal Theory. 9:295–331.

[5] Newman JO . 1993. Beyond “reasonable doubt”. N Y Univ Law Rev. 68(5):979.

[6] Blackstone W . Commentaries on the Laws of England. 3rd ed. Clarendon Press, Oxford, 1786 (4).

[7] 84 FR 63891. 2019.

[8] Boettrich S, Starykh S, NERA Economic Consulting. Recent trends in securities class action litigation: 2018 full-year review. 2019.

[9] Carlsmith KM, Darley JM, Robinson PH. 2002. Why do we punish? Deterrence and just deserts as motives for punishment. J Pers Soc Psychol. 83(2):284–299. 12150228 10.1037/0022-3514.83.2.284

[10] U.S. Department of Justice . Justice expenditure and employment extracts, NCJ 248628. 2015.

[11] Towers W . 2011 update on U.S. tort cost trends. 2012.

[12] Wagner P, Rabuy B, Prison Policy Initiative. Following the money of mass incarceration. 2017.

[13] Cheng E . 2013. Reconceptualizing the burdens of proof. Yale Law J. 122:1254.

[14] Tillers P, Gottfried J. 2006. Case comment- United States v. Copeland, 369 F. Supp. 2d 275 (E.D.N.Y. 2005): a collateral attack on the legal maxim that proof beyond a reasonable doubt is unquantifiable? Law Probab Risk. 5(2):135–157.

[15] Dhami MK, Lundrigan S, Mueller-Johnson K. 2015. Instructions on reasonable doubt: defining the standard of proof and the juror's task. Psychol Pub Policy Law. 21(2):169–178.

[16] Horowitz IA, Kirkpatrick LC. 1996. A concept in search of a definition: the effects of reasonable doubt instructions on certainty of guilt standards and jury verdicts. Law Hum Behav. 20(6):655–670.

[17] Teitcher J, Scurich N. 2017. On informing jurors of potential sanctions. Law Hum Behav. 41(6):579–587. 28816465 10.1037/lhb0000261

[18] Park K, Seong Y, Kim M, Kim J. 2016. Juror adjustments to the reasonable doubt standard of proof. Psychol Crime Law. 22(6):599–618.

[19] Shen FX, Hoffman MB, Jones OD, Greene JD, Marois R. 2011. Sorting guilty minds. N Y Univ Law Rev. 86:1306.

[20] Wichmann FA, Hill NJ. 2001a. The psychometric function: I. Fitting, sampling, and goodness of fit. Percept Psychophys. 63(8):1293. 11800458 10.3758/bf03194544

[21] Thompson WC . 2023. Shifting decision thresholds can undermine the probative value and legal utility of forensic pattern-matching evidence. Proc Natl Acad Sci U S A. 120(41):e2301844120. 37782790 10.1073/pnas.2301844120 PMC10576151

[22] Pearson JM, et al 2018. Modeling the effects of crime type and evidence on judgments about guilt. Nat Hum Behav. 2:856–866. 30931399 PMC6436087

[23] Klein SA . 2001. Measuring, estimating, and understanding the psychometric function: a commentary. Percept Psychophys. 63(8):1421–1455. 11800466 10.3758/bf03194552

[24] Kroll NEA, Yonelinas AP, Dobbins IG, Frederick CM. 2002. Separating sensitivity from response bias: implications of comparisons of yes-no and forced-choice tests for models and measures of recognition memory. J Exp Psychol Gen. 131(2):241–254. 12049242

[25] Kagehiro DK, Stanton WC. 1985. Legal vs. quantified definitions of standards of proof. Law Hum Behav. 9(2):159–178.

[26] Champion DJ . 1989. Private counsels and public defenders: a look at weak cases, prior records, and leniency in plea bargaining. J Crim Justice. 17(4):253–263.

[27] Lederman L . 1999. Which cases go to trial: an empirical study of predictors of failure to settle. Case West Reserve Law Rev. 49(2):315.

[28] McGovern DP, Roach NW, Webb BS. 2014. Characterizing the effect of multidirectional motion adaption. J Vis. 14(13):2. 10.1167/14.13.2 PMC4217536 25368339

[29] Pilly PK, Seitz AR. 2009. What a difference a parameter makes: a psychophysical comparison of random dot motion algorithms. Vision Res. 49:1599–1612. 19336240 10.1016/j.visres.2009.03.019 PMC2789308

[30] Van Wezel RJA, Britten KH. 2002. Motion adaptation in area MT. J Neurophysiol. 88:3469–3476. 12466461 10.1152/jn.00276.2002

[31] Freedman JL, Krismer K, MacDonald JE, Cunningham JA. 1994. Severity of penalty, seriousness of the charge, and mock jurors' verdicts. Law Hum Behav. 18(2):189–202.

[32] Allen RJ, Smiciklas C. 2022. The Law's aversion to naked statistics and other mistakes. Legal Theory. 28(3):179–209.

[33] Hartsough LES . 2020. Effects of legal instructions on behavioral and neural mechanisms of decision-making [doctoral dissertation]: Vanderbilt University, ir.vanderbilt.edu.

