# Supplementary material for: A psychometric approach to decision-making thresholds across legal and societal domains
Source: PNAS Nexus. 2025 Jan 2;4(1):pgae592. doi: 10.1093/pnasnexus/pgae592 (PMC11742129; doi:10.1093/pnasnexus/pgae592)
Supplement: pgae592_Supplementary_Data [file pgae592_supplementary_data.docx]

**
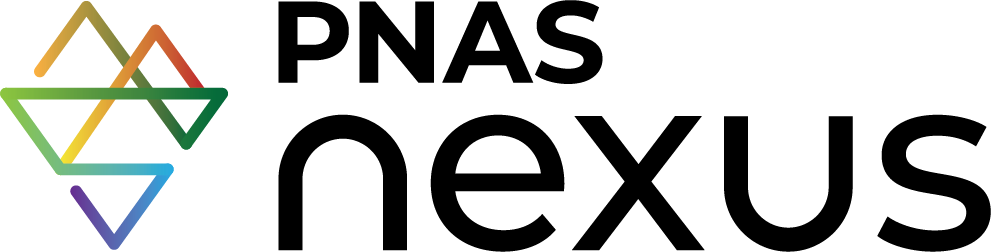
**

A Psychometric Approach to Decision-Making Thresholds Across Legal and Societal Domains

Lauren Hartsough, Matthew Ginther, Edward K. Cheng, and René Marois

Paste Please address correspondence to Lauren Hartsough or René Marois

Email: [laharts12@gmail.com](mailto:laharts12@gmail.com), [rene.marois@vanderbilt.edu](mailto:rene.marois@vanderbilt.edu)

**This PDF file includes:**

Section 1. Experiment 1- Legal vs Non-Legal Contexts

1. Methods
2. Results
3. Full Scenarios

Section 2. Experiment 1A- Scenario Wording Control

1. Methods
2. Results

Section 3. Experiment 1B- Explicitly Civil PoE Context Control

1. Methods
2. Results

Section 4. Experiment 2- Perceptual Context

1. Methods
2. Results

Section 5. Experiment 2A- Single-Trial Perceptual Control

1. Methods
2. Results

Section 6. Experiment 3- Decision Costs

1. Methods
2. Results
3. Full Scenarios

Section 7. Experiment 3A- Decision Cost Severity Ratings Control

1. Methods
2. Results

Supplemental References

**Section 1A. Experiment 1- Legal vs Non-Legal Contexts: Extended Methods**

**Participants.** We recruited 6067 participants (53% male, Mean age=35.25 years) from the United States via Amazon Mechanical Turk (Crump, McDonnell, & Gureckis, 2013). We eliminated 599 participants for failing attention checks about the content of the trial/instructions, yielding a total of 5468 participants included in our analyses. Participants were paid $0.40 for completing the study, which took less than five minutes on average. Past research suggests that 40 responses per group provides strong coverage of bootstrap confidence intervals for psychometric parameters (i.e. intervals more likely to contain the true value; Fründ, Haenel, & Wichmann, 2011). We therefore recruited participants until we reached at least 40 responses per cell (context x objective evidence strength x instruction) after all exclusion criteria had been applied. All participants provided informed consent, and the experimental protocol was approved by the Vanderbilt University Institutional Review Board.

**Task Design.** The experiment was administered using the Qualtrics online survey platform. Each participant read and responded to a single randomly assigned scenario that was one paragraph in length. The task employed a 2 (scenario context: legal or non-legal) x 7 (objective evidence strength; 20%, 40%, 60%, 80%, 95%, 99%, 100%) x 3 (decision criteria instruction; IB, PoE, BaRD) between-subjects design.

Figure S1.1 shows a sample trial (Legal context, drug theft with fingerprint scenario, 80% evidence strength, and BaRD instruction); all the scenarios used are included in Section 1C of the Supplementary Materials. The principal phases of a trial consisted of scenario presentation, criterion instructions, decision response, and finally probability estimation. Participants were first presented with the scenario text on their computer screen. The decision criteria instructions then appeared directly below the scenario on the same screen once participants pressed a button to continue after reading through the scenario. After reading the instructions, participants responded to a question that appeared on the same screen under both the scenario and instructions. Thus, both the scenario and the instructions were available to participants while making their decision. Evaluation of the scenario/instructions and the subsequent decisions were self-paced.

**Scenario Presentation.** Participants were randomly assigned to a single scenario that described either a legal or non-legal context and presented the objective evidence. The subject matter of the **legal scenarios** involved a protagonist, Mark, who may have engaged in conduct that is widely accepted as criminal or civil wrong doing. To cover a broad spectrum of potential legal contexts, the scenarios varied in the fact pattern (stealing prescription drugs, stealing company data, and murder) and in the type of evidence available (video facial recognition, fingerprints, and DNA), thus forming nine possible legal scenarios. The objective evidence strength was presented within the scenario as the level of certainty with which investigators were able to link the available evidence to the protagonist. This evidence strength was communicated with a frequentist measure of probability that varied between subjects across seven possible levels: 20%, 40%, 60%, 80%, 95%, 99%, and 100% (e.g. Investigators concluded with 40% certainty that the DNA found belonged to the protagonist). To increase the realistic interpretation of these probabilities (Wells, 1992; M.G. & R.M, unpublished data), the legal scenarios were crafted so that they described ‘closed systems’ in which the person who committed the offense was a member of a finite group of individuals (for example, the culprit can only be among the individuals aboard a ship). We compared decisions by fact pattern and by evidence type within the legal context and found that decision thresholds were not significantly different between fact patterns or evidence type (Supplementary Figs S1.3-S1.4 and Tables S1.1-S1.2); we therefore collapsed the data across fact pattern and evidence type.

The subject matter of the **non-legal scenarios** eschewed legal or wrong-doing matters and instead described situations that required participants to render a decision about the occurrence of an event in one of five distinct fact patterns. Specifically, participants were tasked to make a judgement about the likelihood of either: a patient developing Huntington’s disease, a stock underperforming in the market, abnormal water temperatures developing in the Pacific Ocean, the occurrence of a petroleum spill, or the presence of electronic spam information. The objective evidence strength was presented as a frequentist probability that the condition or event occurred or would occur, and varied between 20%, 40%, 60%, 80%, 95%, 99%, and 100%. As in the legal context, we compared decisions by fact pattern within the non-legal context (this context did not have different evidence types), and found that decision thresholds did not differ significantly between fact patterns (Supplementary Fig. S1.5 and Table S1.3); we therefore collapsed the data across fact pattern.

**Criterion Instructions.** After reading the scenario, participants pressed a button that made the decision instructions appear just below the scenario. The decision criteria instructions could take one of three forms. The first included no specific criterion language so as to assess the participants’ intrinsic decision criteria in the absence of external guidelines. This condition is referred as the “intuitive belief” (IB) instruction condition. The two other instructions corresponded to the legal burdens of proof of preponderance of the evidence (PoE) and beyond a reasonable doubt (BaRD). These two burdens of proof were excised from pattern jury instructions adopted by federal courts across the country (U.S. District Court N. D. Cal, 2012) and adapted so that they could be applied to both legal and non-legal contexts. Figure S1.2 provides sample decision criteria instructions for legal (top row) and non-legal (middle row) contexts. After reading the instructions participants pressed a button to acknowledge they read and would abide by the instructions, which prompted the Decision Response text to appear below on the screen.

**Decision Response.** Participants selected either “Yes” or “No” in response to a question about their beliefs regarding the scenario. Specifically, those in the intuitive belief condition were asked if they believed that the action or event described in the scenario had or would occur (e.g. “Do you believe that Mark stole the prescription drugs?”; “Do you believe that the patient will develop Huntington’s disease?”). For those in either of the burden of proof instruction conditions, the prompt included the specific instruction language within the question (e.g. “Do you believe by a preponderance of the evidence that Mark stole the prescription drugs?”; “Do you believe beyond a reasonable doubt that the patient will develop Huntington’s disease?”). This language was included to ensure that participants were incorporating the instructions into their subsequent decisions as they were instructed to do. We avoided using words such as “responsible” or “guilty” to probe participants’ beliefs in the legal scenarios in order to keep the prompts comparable across both legal and non-legal contexts, and because pilot data indicated that, for some subjects, these words focused the subjects’ attention on the appropriateness of the consequences (i.e., punishment or monetary liability) that may befall the protagonist.

**Probability Estimation.** After providing a yes/no response in the Decision phase, participants proceeded to a new screen which asked them to provide their own subjective probability for the event occurring (e.g. “What do you believe is the probability that Mark stole the prescription drugs?”; “What do you believe is the probability that the patient will develop Huntington’s disease?”). Participants responded by clicking and dragging a bar along a number line ranging from 0 to 100. We probed the participants’ subjective evidence strength as a measure of the probability they were actually considering when making their decision and to determine the extent to which they believed the information provided in the scenario (i.e. the objective evidence strength). Consistent with previous studies comparing subjective and objective probability estimates (Erev, Wallsten, & Budescu, 1994; Meyniel, Schlunegger, & Dehaene, 2015), participants tended to overestimate the evidence strength for lower levels of the objective evidence while underestimating the strength for higher levels of the objective evidence (Fig. S1.6). We focused our analyses on the subjective evidence strength as this was the probability that participants likely applied when making their decision. However, since the subjective evidence was collected after the participants had already made their decision response, we reanalyzed Experiment 1 using the objective evidence strength to determine whether the results were qualitatively different. That wasn’t the case. Specifically, the decision thresholds for each instruction were not significantly different between subjective and objective evidence, and the decision threshold order across instructions (BaRD>PoE>IB) was the same across both types of evidence. Not surprisingly the objective and subjective evidence functions diverged more at the extremities of the distribution (Fig. S1.7).

Following their response to the probability estimation, participants were prompted to an attention check question on a new screen to determine whether they had carefully read the scenario and instructions. Finally, they provided basic demographic information and were debriefed.

**Statistical Analyses.** Psychometric functions were used to characterize the likelihood of an affirmative response by evidence strength, context, and instruction type. For the legal context, an affirmative response meant that based on the evidence participants believed that the protagonist Mark had performed the action described in the scenario. For the non-legal context, an affirmative response meant that based on the evidence participants believed that the event in the scenario had occurred or was going to occur (for instance that a patient would go on to develop Huntington’s disease). Analyses were completed using the quickpsy package for R version 3.5.3 (Linares & Lopez-Moliner, 2017) together with custom R code.

Psychometric curves for each condition were fit using maximum likelihood methods and the logistic function, which allows the threshold and slope parameters to vary independently of one another (Gilchrist, Jerwood, & Ismaiel, 2005). Our stimuli strength was participants’ subjective probability estimates binned to ensure sufficient responses per level to assess goodness- of-fit (bin size 10, plus bins for 95, 99, and 100; Wichmann & Hill, 2001a). We assessed the goodness-of-fit for each curve by calculating the deviance, as well as a distribution of deviances from our 1000 bootstrap samples (Wichmann & Hill, 2001a; Linares & Lopez-Moliner, 2017). A p value less than alpha (<0.05; deviance not within 95% CI from distribution) indicates that the curve is not a good fit for the data. That all p values were greater than 0.46 indicates that the psychometric functions fit the data well.

We obtained estimates of four psychometric parameters from these curves. The threshold parameter was defined as the value of the evidence strength at which an affirmative response becomes more likely (i.e. value of x when y=0.50), while the slope parameter is the slope of the curve at this threshold. Because some conditions did not reach a lower and/or upper asymptote, we instead use the terms lower and upper bounds to describe the predicted value of y at x=0 and x=100 respectively. We generated 95% confidence intervals for the psychometric curve and parameter estimates using 1000 parametric bootstrap samples of our data (Linares & Lopez- Moliner, 2017; Wichmann & Hill, 2001b).

We then compared these parameter estimates between conditions via planned pairwise comparisons of the effect of instruction type within each context level as well as the effect of context within each level of instruction type. For each pairwise comparison, we generated a distribution of difference scores using the 1000 bootstrap estimations from the two parameters (Linares & Lopez-Moliner, 2017; Wichmann & Hill, 2001b), which allowed us to generate confidence intervals with a Bonferroni correction for multiple comparisons (i.e. CI=1-(0.05/# comparisons)). Confidence intervals that do not contain zero indicate a significant difference between groups. This statistical approach was warranted with the present bootstrapped data because it does not rely on assumptions about the underlying population distribution, in contrast to parametric tests.

**
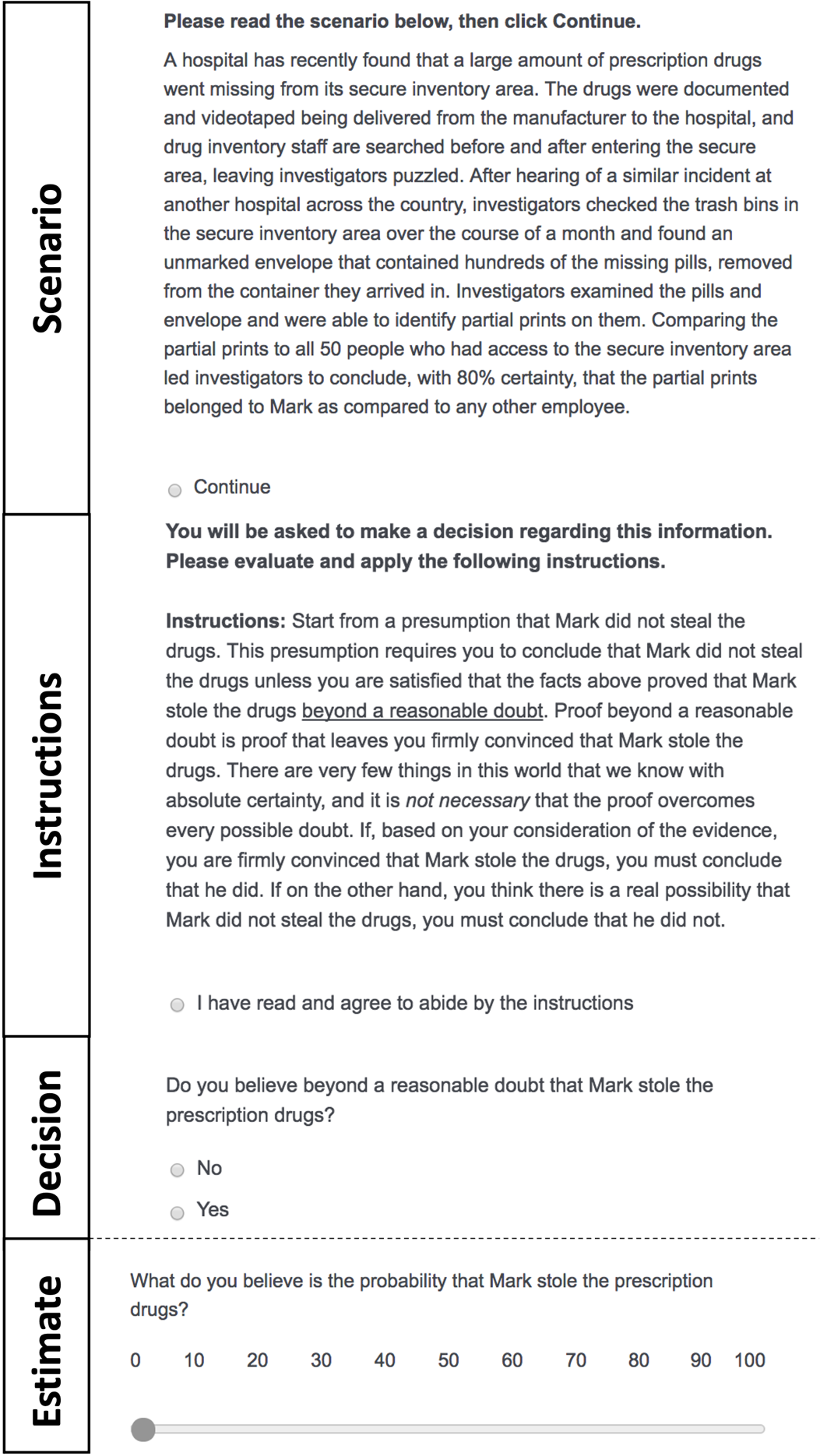
**

**Fig. S1.1** Sample trial as seen by participants in the legal context (prescription drug theft x finger print evidence scenario) with BaRD instruction.


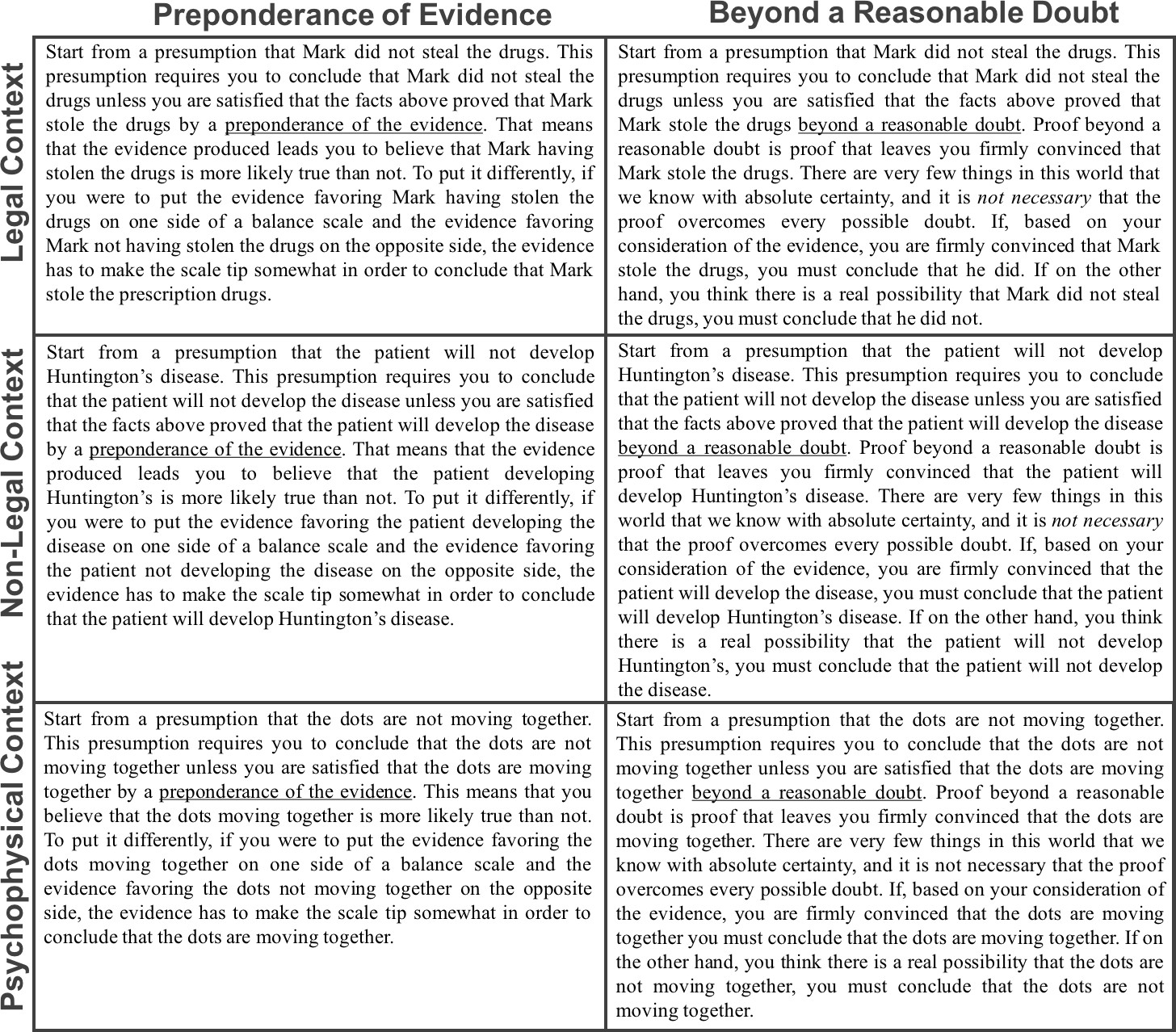


**Fig. S1.2** Sample instructions for preponderance of the evidence (PoE) and beyond a reasonable doubt (BaRD). The top and middle rows show the sample instructions from Experiment 1 for the legal and non-legal context respectively. The bottom row shows the instructions for the psychophysical task in Experiment 2.

**Section 1B. Experiment 1- Legal vs Non-Legal Contexts: Extended Results**

**Table S1.1.** Pairwise differences and Bonferroni-corrected CIs between legal scenario fact patterns in Experiment 1. Confidence intervals that do not contain 0 indicate a significant difference (indicated by *).


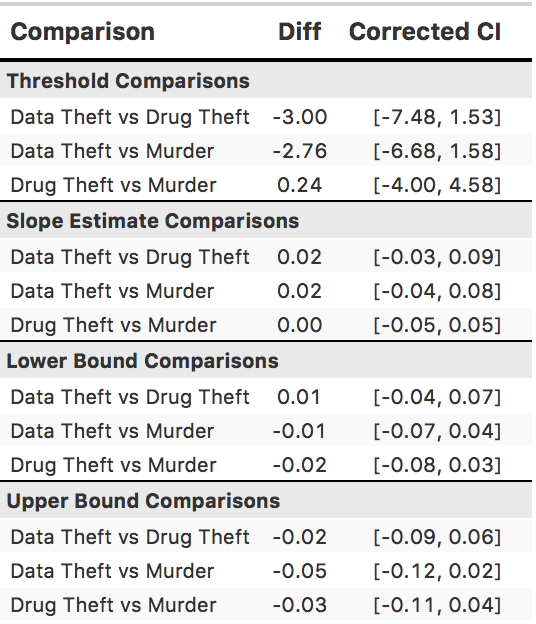

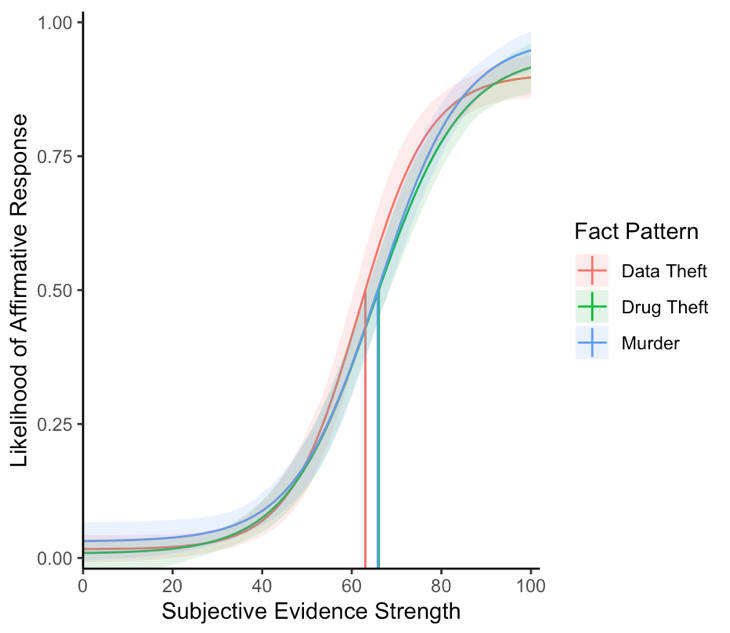


**Fig. S1.3.** Likelihood of an affirmative response by subjective evidence strength and fact pattern for legal scenarios. Shaded regions are 95% confidence intervals estimated via 1000 bootstrap samples. Decision thresholds are marked with vertical lines.

**Table S1.2.** Pairwise differences and Bonferroni-corrected CIs between legal scenario evidence types in Experiment 1. Confidence intervals that do not contain 0 indicate a significant difference (indicated by *).


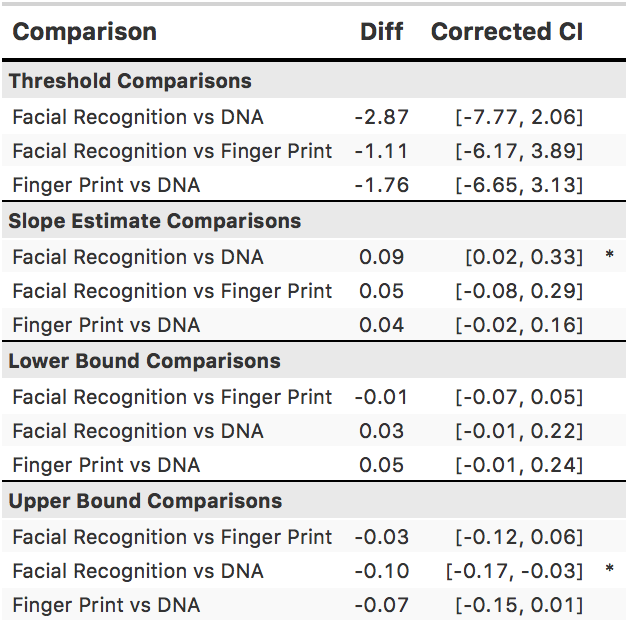

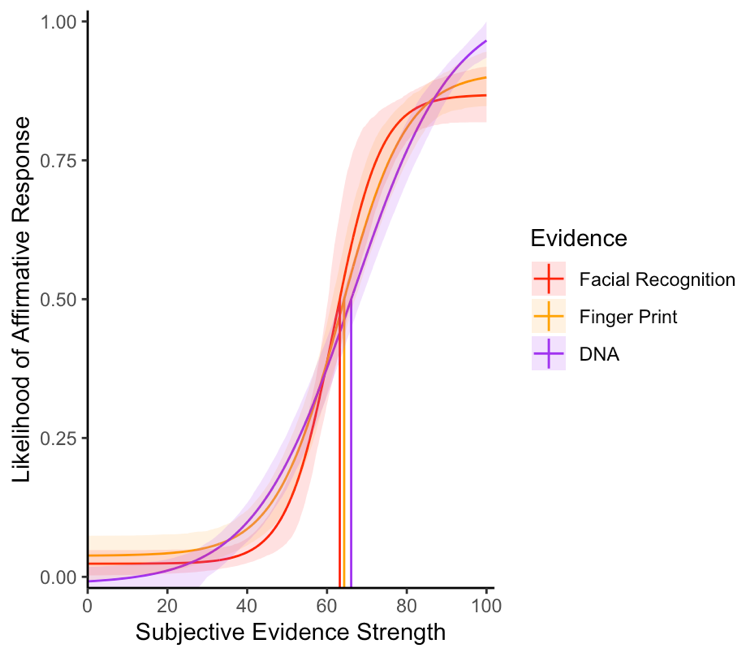


**Fig. S1.4.** Likelihood of an affirmative response by subjective evidence strength and evidence type for legal scenarios. Shaded regions are 95% confidence intervals estimated via 1000 bootstrap samples. Decision thresholds are marked with vertical lines.


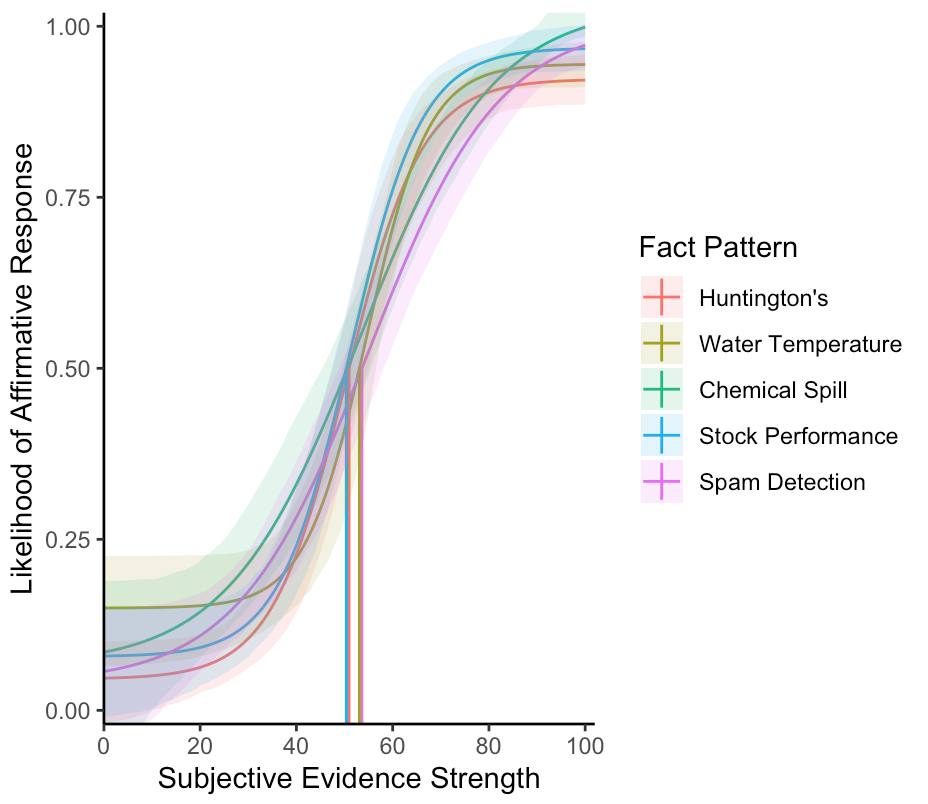


**Fig. S1.5.** Likelihood of an affirmative response by subjective evidence strength and scenario content for non-legal scenarios. Shaded regions are 95% confidence intervals estimated via 1000 bootstrap samples. Decision thresholds are marked with vertical lines.

**Table S1.3.** Pairwise differences and Bonferroni-corrected CIs between non-legal scenario fact patterns in Experiment 1. Confidence intervals that do not contain 0 indicate a significant difference (indicated by *).


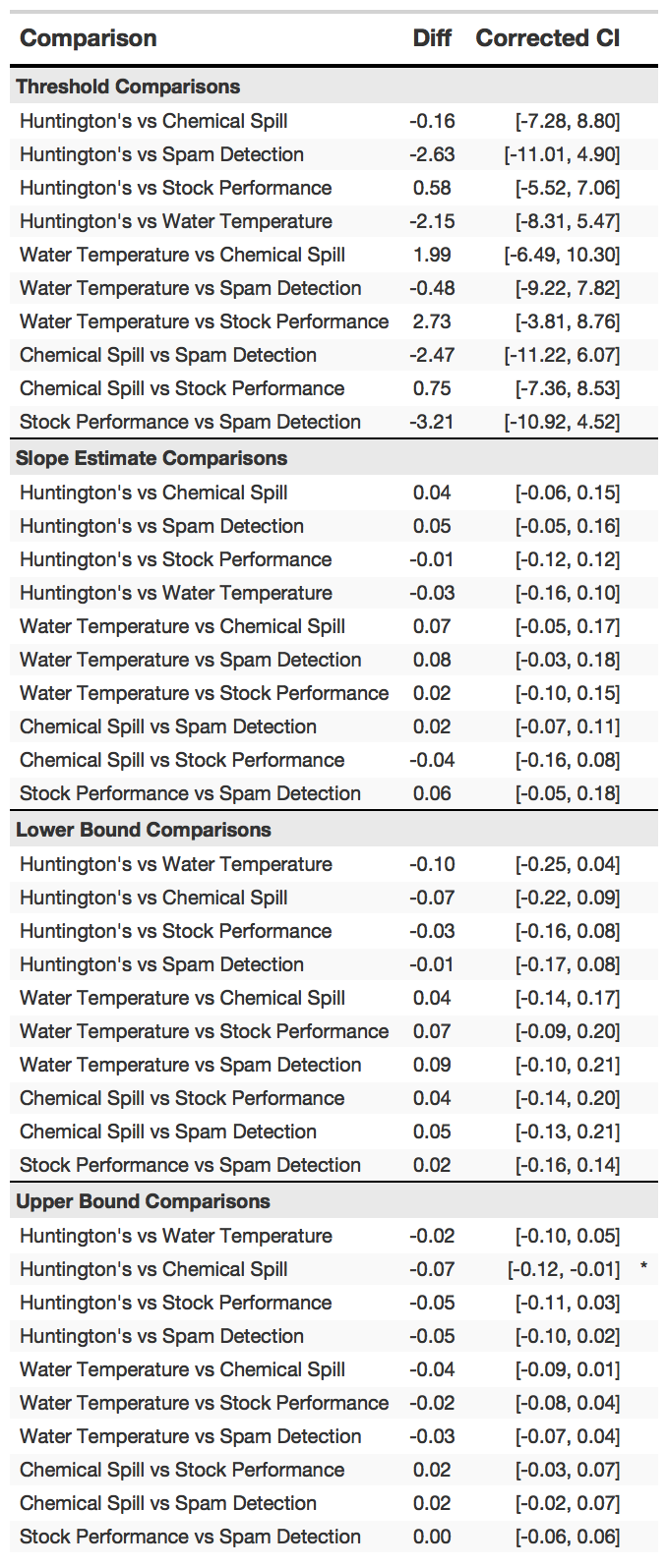

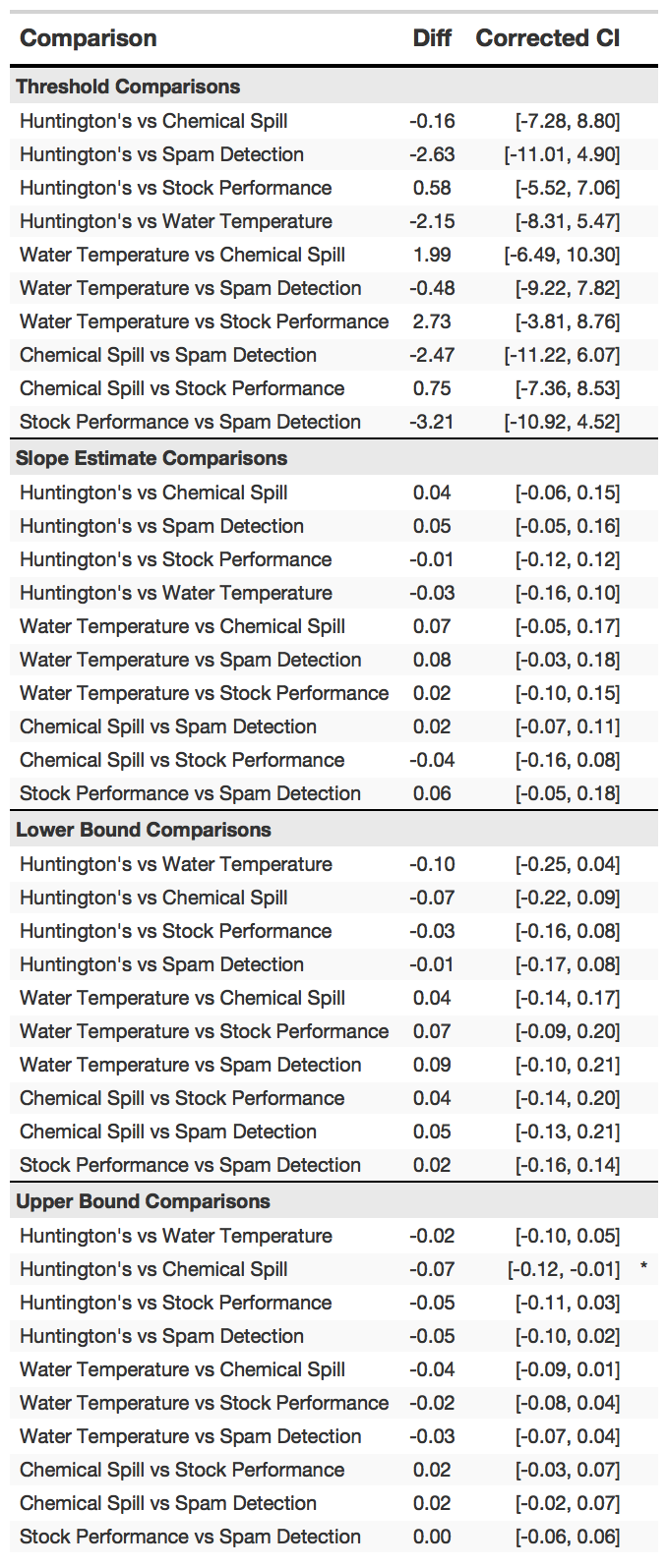


**
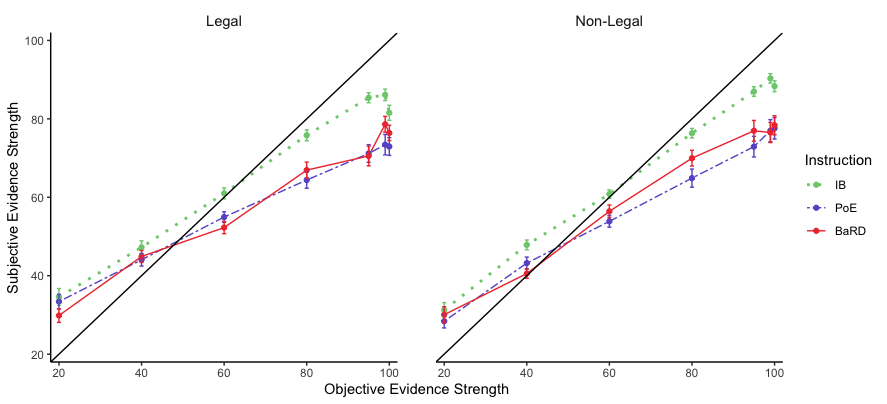
**

**Fig. S1.6.** Mean estimated subjective evidence strength by objective evidence strength and instruction type for legal and non-legal contexts. 95% confidence intervals estimated via 1000 bootstrap samples. The black line represents the “ideal” in which objective evidence strength = subjective evidence strength.


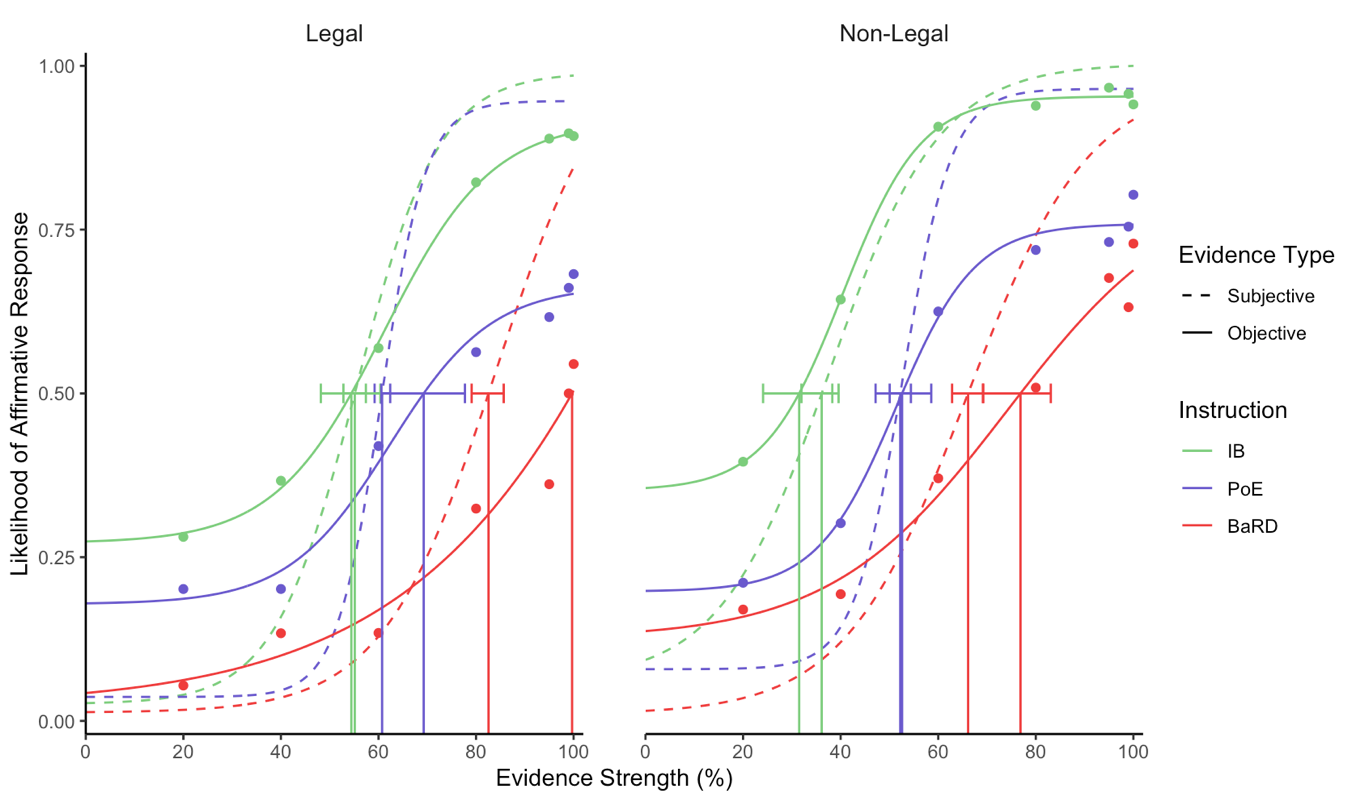


**Fig. S1.7.** Likelihood of an affirmative response by objective evidence (solid lines; value used in the scenario), subjective evidence (dashed lines; value given by subjects), instruction, and context for Experiment 1. These results are qualitatively the same using subjective or objective evidence strength.

**Table S1.4.** Estimates and 95% CIs for the psychometric parameters in Experiment 1.


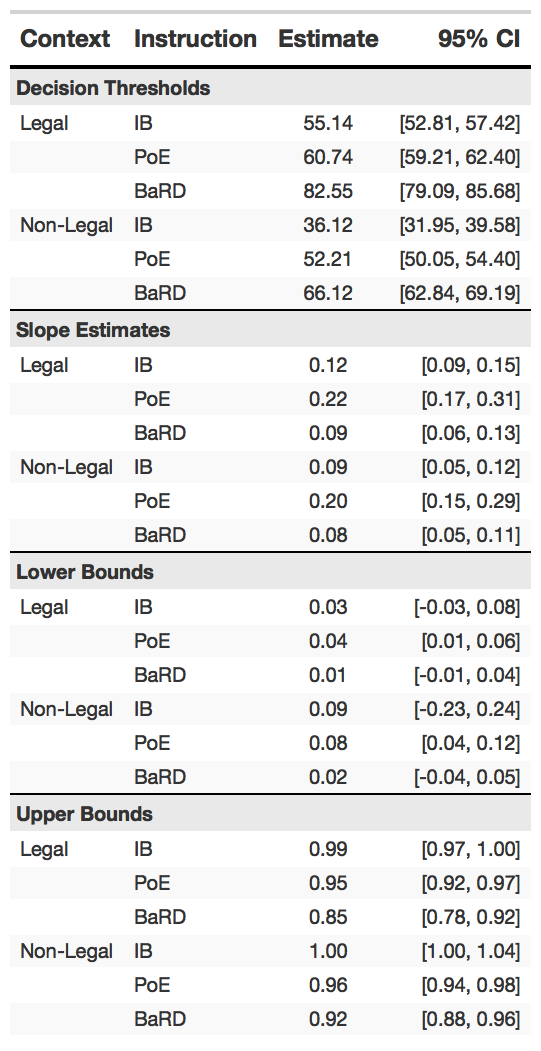


**Table S1.5.** Pairwise differences and Bonferroni-corrected CIs between conditions (Instruction x Context) in Experiment 1. Confidence intervals that do not contain 0 indicate a significant difference (indicated by *)


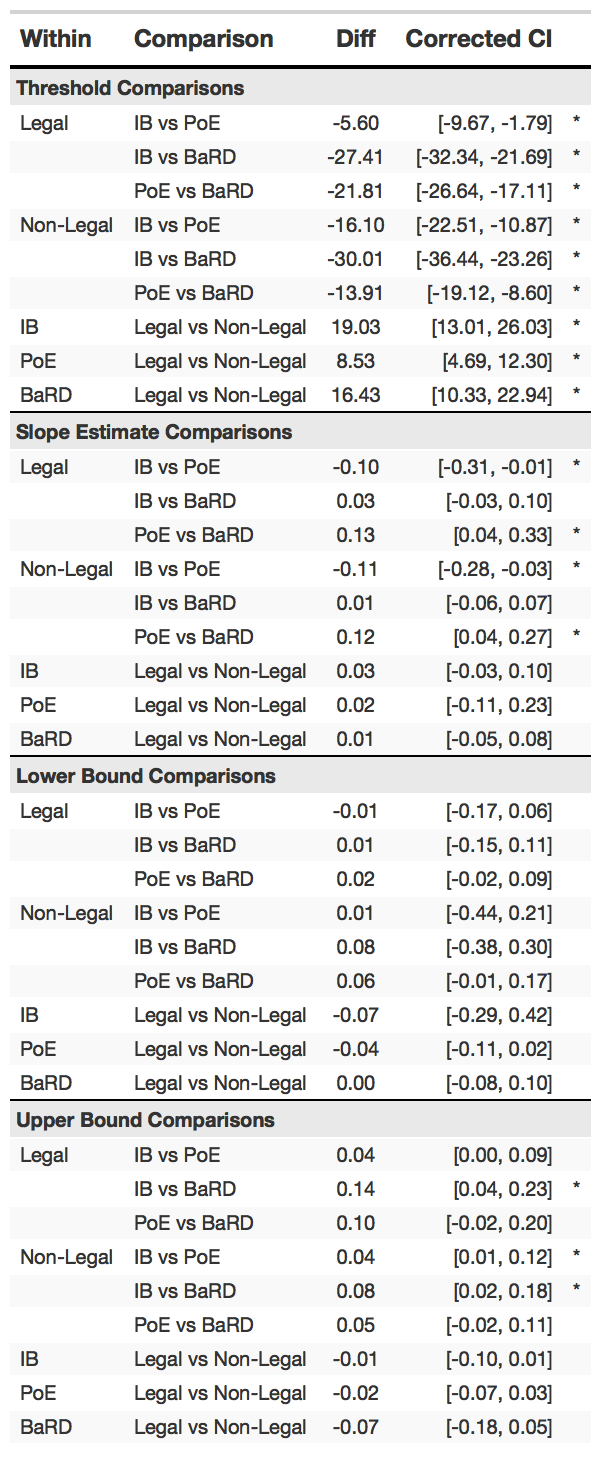


**Section 1C. Experiment 1- Legal vs Non-Legal Contexts: Full Scenarios**

Legal: Stealing company data x Facial recognition

A company recently identified a security breach concerning some of its proprietary data. The data was downloaded from a server room in the secure wing of one of their office buildings. The secure wing is under 24-hour surveillance and can only be accessed by presenting ID and biometric information (a thumbprint) at the only entrance and exit. Investigators examined the security tapes and found grainy footage showing that only one individual–a white male employee–entered the server room at the time the breach occurred. At the time of the breach, 50 white males were recorded as being in the secure wing of the building. A facial recognition analysis comparing the person in the video with pictures of all 50 white males who were in the secured area at the time of the breach led investigators to conclude, with [Objective Evidence] % certainty, that the person in the video was Mark as compared to anyone else in the office.

Legal: Stealing company data x Finger prints

A company recently identified a security breach concerning some of its proprietary data. The data was downloaded from a server room in the secure wing of one of their office buildings. The secure wing is under 24-hour surveillance and can only be accessed by presenting ID and biometric information (a thumbprint) at the only entrance and exit. At the time of the breach, 50 people were recorded as being in the secure wing of the building. Investigators examined the server and were able to identify partial prints on the inside of a piece of plastic siding that was broken off in order to access the server’s port. Because the fingerprint was from the inside of the server casing it could only have been left by the person responsible for the data breach. Comparing the partial prints to all 50 employees who were in the secure wing at the time of the breach led investigators to conclude, with [Objective Evidence] % certainty, that the partial prints belonged to Mark as compared to anyone else in the office.

Legal: Stealing company data x DNA

A company recently identified a security breach concerning some of its proprietary data. The data was downloaded from a server room in the secure wing of one of their office buildings. The secure wing is under 24-hour surveillance and can only be accessed by presenting ID and biometric information (a thumbprint) at the only entrance and exit. At the time of the breach, 50 people were recorded as being in the secure wing of the building. Investigators examined the server and found a small amount of degraded blood on a sharp broken edge of the side panel, left when the perpetrator pried the panel off to access the server’s port. Because the evidence was from the inside of the server casing it could only have been left by the person responsible for the data breach. Comparing the DNA in the blood with DNA samples from all 50 individuals in the secured area at the time of the breach led investigators to conclude, with [Objective Evidence] % certainty, that the blood on the broken server panel came from Mark as compared to anyone else in the office.

Legal: Stealing prescription drugs x Facial recognition

A hospital has recently found that a large amount of prescription drugs went missing from its secure inventory area. The drugs were documented and videotaped being delivered from the manufacturer to the hospital, and drug inventory staff are searched before and after entering the secure area, leaving investigators puzzled. After hearing of a similar incident at another hospital across the country, investigators regularly checked the trash bins in the secure inventory area over the course of a month and found an unmarked envelope that contained hundreds of the missing pills, removed from the container they arrived in. Investigators examined the security tapes and found grainy footage showing a white male employee placing the unmarked envelope in the trash bin. A facial recognition analysis comparing the person in the video with pictures of all 50 white males who had access to the secure inventory area led investigators to conclude, with [Objective Evidence] % certainty, that the person in the video was Mark as compared to any other employee.

Legal: Stealing prescription drugs x Finger prints

A hospital has recently found that a large amount of prescription drugs went missing from its secure inventory area. The drugs were documented and videotaped being delivered from the manufacturer to the hospital, and drug inventory staff are searched before and after entering the secure area, leaving investigators puzzled. After hearing of a similar incident at another hospital across the country, investigators checked the trash bins in the secure inventory area over the course of a month and found an unmarked envelope that contained hundreds of the missing pills, removed from the container they arrived in. Investigators examined the pills and envelope and were able to identify partial prints on them. Comparing the partial prints to all 50 people who had access to the secure inventory area led investigators to conclude, with [Objective Evidence] % certainty, that the partial prints belonged to Mark as compared to any other employee.

Legal: Stealing prescription drugs x DNA

A hospital has recently found that a large amount of prescription drugs went missing from its secure inventory area. The drugs were documented and videotaped being delivered from the manufacturer to the hospital, and drug inventory staff are searched before and after entering the secure area, leaving investigators puzzled. After hearing of a similar incident at another hospital across the country, investigators checked the trash bins in the secure inventory area over the course of a month and found an unmarked envelope that contained hundreds of the missing pills, removed from the container they arrived in. Investigators examined the envelope and were able to recover degraded saliva that was used to seal it shut. Comparing the DNA in the saliva to DNA samples from all 50 people who had access to the secure inventory area led investigators to conclude, with [Objective Evidence] % certainty, that the saliva came from Mark as compared to any other employee.

Legal: Murder x Facial recognition

While at sea, the body of a young female crewmember of the tanker was found raped and murdered in a storage container after having been missing for 3 days. There were no eyewitness accounts and not enough physical evidence to be of use. The storage container is accessible to everyone on the tanker. Investigators examined the security tapes from the area of the ship where the container is located and found grainy footage showing that only one individual–a white male crewmember–accessed the storage container during that three-day period. A facial recognition analysis comparing the person in the video with pictures of all 50 white males on the ship led investigators to conclude, with [Objective Evidence] % certainty, that the person in the video was Mark as compared to anyone else on board.

Legal: Murder x Finger prints

While at sea a young female crewmember of the tanker was found raped and murdered after having been missing for 3 days. There were no eyewitness accounts or security cameras, and there was not enough physical evidence to be of use. The knife used to commit the murder was found with the body, covered with the victim’s blood. Investigators examined the knife and were able to identify partial prints in the dried blood on the handle, which were the only fingerprints left on the entire knife. Comparing the partial prints to all 50 males onboard the ship led investigators to conclude, with [Objective Evidence] % certainty, that the partial print belonged to Mark as compared to anyone else on board.

Legal: Murder x DNA

While at sea a young female crewmember of the tanker was found raped and murdered after having been missing for 3 days. There were no eyewitness accounts and the limited number of security cameras on board the ship were not helpful in the identification. There is little physical evidence, but some degraded semen was recovered from the body and the DNA matches the DNA collected from a piece of hair found tangled in the rope used to bind the victim’s arms. Comparing the DNA samples from the semen and hair to DNA samples from all 50 males onboard the ship led investigators to conclude, with [Objective Evidence] % certainty, that the semen and hair came from Mark as compared to anyone else on board the ship.

Non-Legal: Huntington’s disease

A genetic test on a patient reveals that a section of their DNA contains 36 repeats of the ‘CAG’ sequence. When patients are found to have this many repeats of the ‘CAG’ sequence it can be concluded, with [Objective Evidence] % certainty, that they will develop Huntington’s disease.

Non-Legal: Stock performance

An analysis of a stock price history shows that the stock’s long-term moving average broke above its short-term moving average. When this occurs it can be concluded, with [Objective Evidence] % certainty, that the stock will underperform the market in the near future.

Non-Legal: Water temperature

Last year, meteorologists detected a premature weakening of the pacific trade winds. When this is observed it can be concluded, with [Objective Evidence] % certainty, that above average water temperatures will develop in the Pacific Ocean.

Non-Legal: Spam packet

A network administrator detects an incoming network packet that shows five indicators consistent with spam. Based on these five indicators it can be concluded, with [Objective Evidence] % certainty, that the incoming network packet is spam.

Non-Legal: Petroleum spill

A local agency is called to investigate a chemical smell emerging from a local river. They detect petroleum in the water at a level of 10 mg/L. When this level of petroleum is detected it can be concluded, with XX% certainty, that there has been a petroleum spill.

**Section 2A. Experiment 1A- Scenario Wording Control: Extended Methods**

To determine whether the greater stringency in the legal versus non-legal context can be explained by a difference in scenario language, we ran a control experiment in which we modified our legal scenarios to match the language in the non-legal scenarios so as to directly link the evidence strength to the wrongdoing that participants rendered a judgment on.

**Participants.** We recruited 2993 new participants via Amazon Mechanical Turk. After excluding 452 individuals who failed the attention check, 2541 participants were included in the final analyses which gave us a number of responses for each psychometric function comparable to those in the original experiment with at least 40 responses per cell after all exclusions.

**Task Design.** All participants responded to a single modified legal scenario randomly assigned out of 9 possible scenarios, as in Experiment 1 (3 fact patterns x 3 types of evidence). For these scenarios, the final sentence of the text was modified to present the strength of the objective evidence as the level of certainty that Mark committed the wrongdoing, in order to match the language in the non-legal scenarios (c.f. example below to that of Experiment 1). The experimental design, including the decision criteria instructions and prompts were otherwise identical to those used in the original experiment.

Sample modified control-legal scenario: Prescription drug theft x DNA evidence

A hospital has recently found that a large amount of prescription drugs went missing from its secure inventory area. The drugs were documented and videotaped being delivered from the manufacturer to the hospital, and drug inventory staff are searched before and after entering the secure area, leaving investigators puzzled. After hearing of a similar incident at another hospital across the country, investigators checked the trash bins in the secure inventory area over the course of a month and found an unmarked envelope that contained hundreds of the missing pills, removed from the container they arrived in. Investigators examined the envelope and were able to recover degraded saliva that was used to seal it shut. Comparing the DNA in the saliva to DNA samples from all 50 people who had access to the secure inventory area it can be concluded, with [Objective Evidence Strength] % certainty, that Mark stole the prescription drugs.

**Statistical Analyses.** All statistical analyses were identical to those used in the Experiment 1.

**Section 2B. Experiment 1A- Scenario Wording Control: Extended Results**

Comparing the legal-control context to the legal and non-legal contexts within instruction type (Fig. S2.1), the legal-control context fell between the legal and non-legal contexts such that decisions were more lenient than the original legal context but more stringent than the non-legal context (Table S2.1). This was most clearly borne out in the legal-control decision thresholds which were between those of the non-legal and legal contexts for all instruction types. Pairwise comparisons showed that the legal-control threshold was significantly different from both original contexts for IB and BaRD. The same trend was present for the PoE instruction though it did not reach significance. Within the legal-control thresholds we observed the same finding as in Experiment 1; IB was significantly more lenient than PoE and BaRD, and PoE was significantly more lenient than BaRD. Furthermore, the legal-control PoE threshold was significantly greater than the prescribed 50% definition (56.80, 95% CI [53.86, 59.51], as in the original legal context.

This suggests that presenting the legal scenarios using language that matched that of the non-legal scenarios did result in less stringent legal decisions, but not to the extent that it can fully account for differences between the legal and non-legal contexts. This suggests that the more stringent decisions in the legal context are due, at least in part, to properties specific to the legal domain.

**
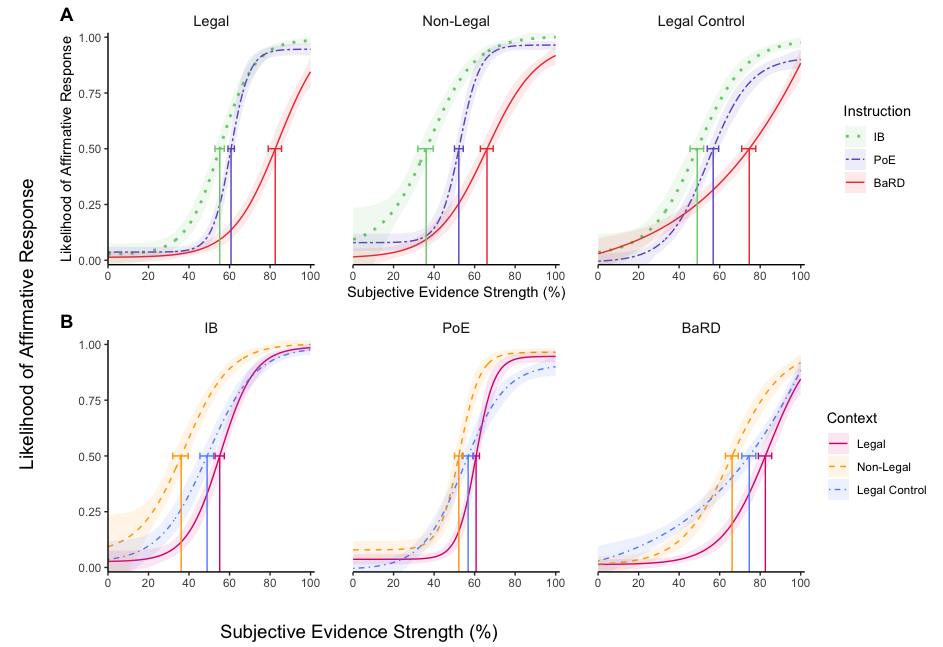
**

**Fig. S2.1.** Likelihood of an affirmative response by subjective evidence strength. Shaded regions are 95% confidence intervals estimated via 1000 bootstrap samples. Decision thresholds are marked with vertical lines and 95% error bars. 1A shows the effect of instruction within each context, 1B shows the effect of context within each instruction.

**Table S2.1.** Pairwise differences and Bonferroni-corrected CIs by context (Experiment 1: Original Legal, Non-Legal; Experiment 1A: Legal Control) and instruction type. Confidence intervals that do not contain 0 indicate a significant difference (indicated by *).

**
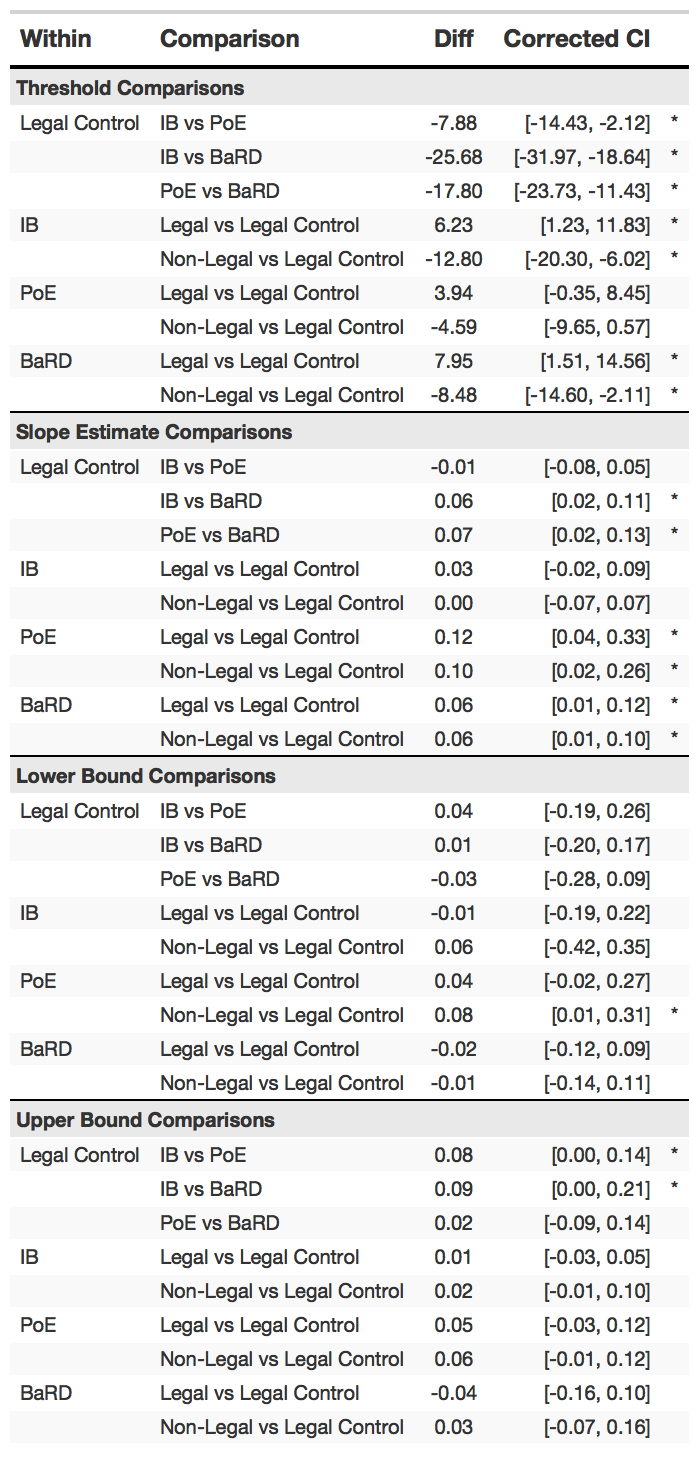
**

**Section 3A. Experiment 1B- Explicitly Civil PoE Context Control: Extended Methods**

To determine whether the overly stringent application of the PoE standard in the legal context can be explained by participants inferring a criminal context, we ran a control experiment in which participants responded to the PoE instruction for a legal scenario in an explicitly civil context (i.e. protagonist faces litigation for wrong-doing).

**Participants.** We recruited 1057 new participants via Amazon Mechanical Turk. After excluding 214 individuals who failed the attention check, 843 participants were included in the final analyses which gave us a number of responses for the psychometric function comparable to those in the original experiment with at least 40 responses per cell after all exclusions.

**Task Design.** All participants responded to a single modified legal scenario randomly assigned out of 9 possible scenarios, as in the original experiment (3 fact patterns x 3 types of evidence). For these scenarios, we added a sentence immediately after the end of the scenario that explicitly indicated a civil context (e.g. “Mark is being sued in civil court for monetary damages related to the [data theft/prescription drug theft/murder]”). The decision criteria instruction was always PoE for this control experiment. The experimental design was otherwise identical to the original experiment.

**Statistical Analyses.** All statistical analyses were identical to those used in Experiment 1.

**Section 3B. Experiment 1B- Explicitly Civil PoE Context Control: Extended Results**

An explicitly civil context did not change the overly stringent application of the PoE instruction within the legal domain (Fig. S3.1). Specifically, the PoE Civil decision threshold was not significantly different from that of the PoE legal threshold in Experiment 1 and was significantly greater than both the prescribed 50% threshold (59.78, 95% CI[57.17, 62.20]) and the PoE non-legal threshold.

Presenting the PoE instruction in an explicitly civil legal context did not result in a more lenient application of the instruction compared to Experiment 1. This suggests that the overly stringent application of the PoE standard is not due to participants inferring a criminal context, and provides further evidence that the more stringent decisions observed in response to legal versus non-legal scenarios are due to specific aspects of the legal context.


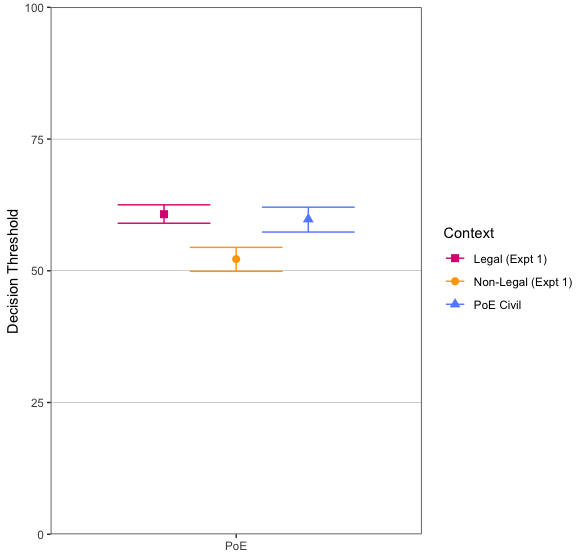


**Fig. S3.1.** Subjective evidence strength at the decision thresholds for the PoE instruction by context for Experiment 1 and the explicitly civil PoE control.

**Section 4A. Experiment 2- Perceptual Context: Extended Methods**

**Participants.** We recruited 72 participants to complete the experiment (26 males, mean age=20.45 years) in the laboratory. All participants provided informed consent, and the experimental protocol was approved by the Vanderbilt University Institutional Review Board. Participants provided basic demographic information and were debriefed at the end of the study. They received either course credit for an introductory psychology course or were paid $12 per hour for completing the task, which took approximately 30 minutes. Twenty-four of the participants emanated from a pilot study in which participants completed 3 blocks, one of each instruction type, but always beginning with IB followed by PoE and BaRD in random order. As we observed an order effect for the presentation of the two legal standards in the pilot study, we chose to present only a single instruction block for the other 48 participants. We included the IB block data from the 24 participants who completed the pilot study because they all completed the IB block first and were not aware of the PoE and BaRD instructions until after they had completed the first (IB) block. The results presented below are qualitatively similar when excluding these 24 participants. One participant was excluded due to their needing to leave before completing the entire task, leaving 71 for the analyses described below.

**Task Design.** . Participants completed a random dot motion task in which they were asked to report whether or not they perceived coherent motion within a set of moving dots, with each participant assigned to one particular instruction type (IB, PoE, or BaRD). The task thus employed a 3 (decision criteria instruction, between-subjects: IB, PoE, BaRD) x 6 (evidence strength, within- subjects; 0%, 20%, 40%, 60%, 80%, 100%) design. The evidence strength corresponded to the percent coherence of the dots, or the proportion of dots moving together in the same direction. Participants completed 30 trials at each coherence level in random order for a total of 180 trials.

**Dot Task Parameters.** The task was presented on a computer screen using custom code via Psychtoolbox Version 3 for MATLAB R2017. Each trial consisted of a random dot motion display. Fifty white dots, each 8 x 8 pixel squares, were presented on a black background within an invisible circular aperture with a 12-degree diameter centered on the screen. Dots were randomly distributed within the circular aperture at the beginning of each trial. The evidence strength/coherence level was randomly assigned for each trial; the proportion of dots moving in the same direction were assigned to move either up or down while the remaining dots were assigned a random direction of movement. Dots moved 5 degrees per second in their assigned direction and had a limited lifetime of 10 frames with a frame rate of 60 Hz. Each dot was assigned a random starting frame value between 0 and 9, and jumped to a new random position within the aperture once this value reached 10, which prevented subjects from tracking the motion of individual dots. Dots that moved outside of the aperture were similarly assigned to a new random position within the aperture to ensure that dot density remained constant.

Each random dot motion trial was displayed for 500 milliseconds followed by a 10 second response window with the question prompt (described below). Once participants responded, there was a 1 second inter-trial interval during which a 0.5° white fixation square appeared in the center of the screen, after which the next trial commenced. Pilot data indicated that these task parameters allowed subjects to reliably identify the 0% and 100% coherence trials while struggling with the intermediary strength levels.

**Task Instructions.** Participants first received verbal instructions describing the task. They were told that they would view a set of moving dots on each trial and would be asked if they believed the dots were moving together. “Moving together” was defined as being able to perceive coherent motion within the dot aperture, either up or down. Participants were told that on each trial they might see all, some, or none of the dots moving together, and that not all the dots had to move in the same direction for them to perceive coherent motion. These verbal instructions were then reiterated in writing on the computer screen at the start of the task for participants to read at their own pace.

**Practice Trials.** Prior to completing the experimental block, participants completed a set of practice trials in order to adapt to the task. Specifically, they first viewed a trial with 100% coherence and were explicitly told that this was a sample of all the dots moving together, followed by a trial with 0% coherence and a statement that this sample represented a case where none of the dots were moving together in the same direction except by chance. Participants then completed 10 additional practice trials with no feedback for a total of 12 practice trials, such that they viewed each coherence level twice (presented in random order) prior to the experimental block.

**Decision Criteria Instructions.** Participants were randomly assigned to one of the three decision criteria instruction levels (IB, PoE, BaRD). Those in the IB condition received no additional instructions after the initial description of the task and practice trials. Participants in the PoE and BaRD conditions read the burden of proof instructions on the screen immediately after completing the practice trials. Figure S1.2 (bottom row) includes the specific language used for both instructions for the dot motion task.

**Subject Responses.** After each trial, participants were asked to provide a yes/no response as to whether or not they believed that the dots were moving together (i.e. “Do you believe that the dots were moving together?”; “Do you believe by a preponderance of the evidence that the

dots were moving together?”; “Do you believe beyond a reasonable doubt that the dots were moving together?”). Participants responded using the keyboard, with “f” for yes and “j” for no.

Note that unlike in Experiment 1, participants were not probed on their subjective probability estimation for the simple fact that they weren’t informed about the objective evidence on a trial-by-trial basis as they were before. Informing them of that evidence would have rendered the dot motion coherence task meaningless as a low-level perceptual counterpart to the high-level cognitive tasks of Experiment 1 as the participants would have used the stated percent coherence of the dots in their judgment instead of solely relying on their perception. Thus, only objective evidence was used in this experiment. As mentioned earlier, the difference between objective and subjective evidence is quantitative, not qualitative (see Fig. S1.7). That is to say, the same relationships across decision parameters exist for both types of evidence.

**Statistical Analyses.** The methods used to generate the psychometric curves and the subsequent statistical analyses were identical to those used in Experiment 1. We assessed the goodness-of-fit using the deviance and distribution of bootstrap deviances. All p values were greater than 0.23 indicating that the curves were a good fit for the data. Pairwise comparisons were again performed by generating a distribution of difference scores using the bootstrap estimations from the two parameters (Linares & Lopez-Moliner, 2017, Wichmann & Hill, 2001b) to generate confidence intervals with a Bonferroni correction for multiple comparisons (i.e. CI=1-(0.05/# comparisons)). Confidence intervals that do not contain zero indicate a significant difference between groups.

**Section 4B. Experiment 2- Perceptual Context: Extended Results**

**Table S4.1.** Estimates and 95% CIs for the psychometric parameters for each condition in Experiment 2.


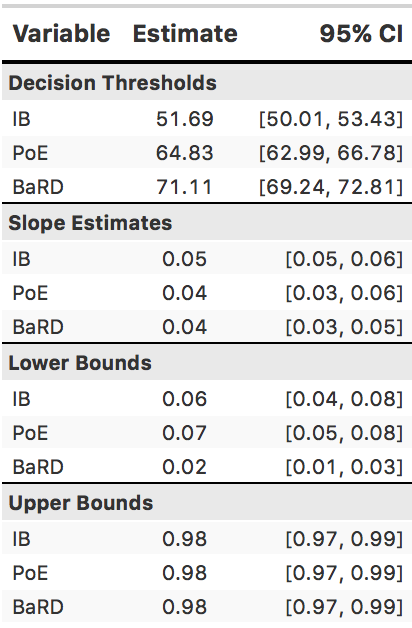


**Table S4.2.** Pairwise differences and Bonferroni-corrected CIs between instructions in Experiment 2. Confidence intervals that do not contain 0 indicate a significant difference (indicated by *)


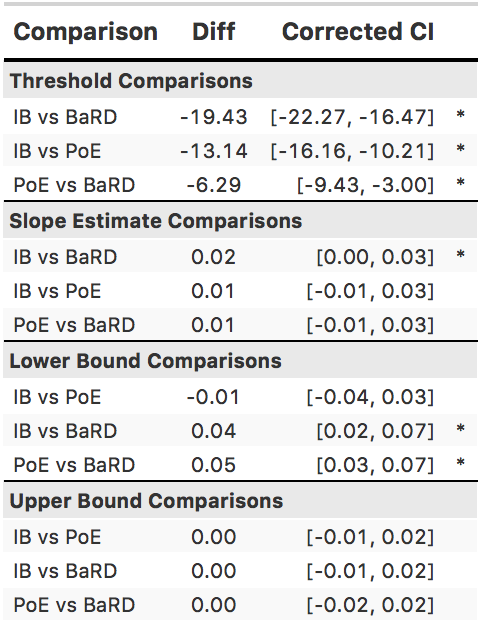


**Table S4.3.** Pairwise differences and Bonferroni-corrected CIs between the legal context in Experiment 1 and Experiment 2, by instruction. Confidence intervals that do not contain 0 indicate a significant difference (indicated by *).


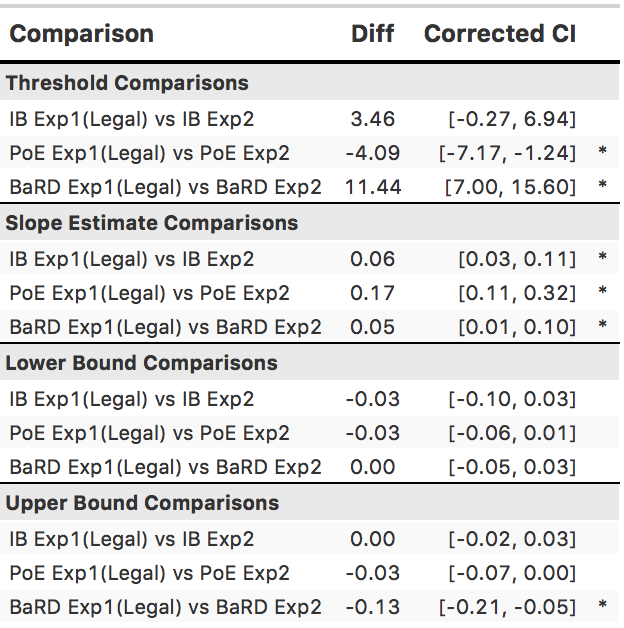


**Table S4.4.** Pairwise differences and Bonferroni-corrected CIs between the non-legal context in Experiment 1 and Experiment 2, by instruction. Confidence intervals that do not contain 0 indicate a significant difference (indicated by *).


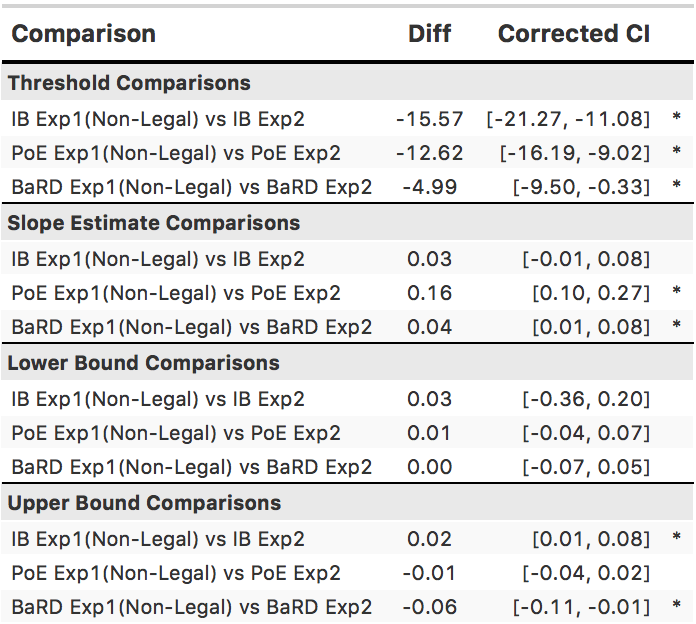


**Section 5A. Experiment 2A- Single-Trial Perceptual Control: Extended Methods**

In this experiment we combined the single-trial approach of Experiment 1 with the random dot motion task of Experiment 2. Participants were therefore exposed to a single decision criteria instruction type and responded to a single dot-motion task trial.

**Participants.** We recruited 1212 participants from the United States via Amazon Mechanical Turk (56% male, mean age=36.82 years). We excluded 18 participants who indicated that they experienced technical issues and were unable to view all of the clips. We further excluded those who did not correctly answer the two attention check trials, leaving a total of 786 participants in subsequent analyses. As in Experiment 1, this allowed us to have forty participants per condition after all exclusions were applied. Participants received $0.40 for completing the task, which took less than five minutes on average. All participants provided informed consent, and the experimental protocol was approved by the Vanderbilt University Institutional Review Board. Participants provided basic demographic information and were debriefed at the end of the study.

**Task Design.** Participants completed a single experimental trial of the random dot motion task described in Experiment 2. The task employed a 3 (decision criteria instruction; IB, PoE, BaRD) x 6 (evidence strength; 0%, 20%, 40%, 60%, 80%, 100%) between-subjects design. As in Experiment 2, the evidence strength corresponded to the percent coherence of the dots, or the proportion of dots moving together in the same direction.

The experiment was administered using the Qualtrics online survey platform. Participants were required to use a computer to complete the survey- the survey screened out individuals who were using a mobile device. This was done to ensure that participants could successfully view the dot stimuli, which were presented as video clips. Participants began by reading the same general task instructions presented in Experiment 2 that described the goal of identifying coherent motion within the dots. Participants completed practice trials before completing a single experimental trial with random assignments to the decision criteria instruction, the coherence level (evidence strength), and the direction of the coherent motion.

**Practice Trials.** Prior to the experimental trial, participants completed 12 practice trials to acclimate them to the task and allow them to see the full range of motion possible. As in Experiment 2, the first trial was an example of 100% coherence with explicit instruction that it was a sample of all of the dots moving together and the second trial explicitly presented a sample with 0% coherence. The responses to these two trials additionally served as attention checks as participants were told beforehand whether none or all of the dots were moving together. The remaining 10 trials were presented in random order such that participants viewed two samples of each level of the evidence strength over the course of the practice trials. Participants provided a yes/no response for each practice trial on a new page to indicate if they believed there was coherent motion.

**Decision Criteria Instructions.** Following the practice trials, participants in the PoE and BaRD instruction conditions received the additional legal standard instructions to apply to their decision which were identical to those used in Experiment 2 (see bottom row of Fig. S1.2). Participants in the IB condition received no additional instruction following the practice trials.

**Dot Task Parameters.** The dot task parameters were identical to those used in Experiment 2. The trials were presented as video clips that were 500 milliseconds each in length. Participants were instructed to view each of the video clips only once.

**Subject Responses.** Participants provided a yes/no response as to whether or not they believed that the dots were moving together at the end of each practice trial and at the end of the single experimental trial. Participants who responded “yes” to perceiving coherent motion were also asked to report whether they perceived the dots moving up or down (though the latter report only served to show that participants were more likely to report the direction incorrectly for lower levels of coherence, suggesting that they were guessing under these conditions).

After completing the experimental trial, participants were asked whether they had experienced any technical issues in viewing the video clips for the trials. They then provided demographic information and were debriefed.

**Statistical Analyses.** Statistical analyses were identical to those used in Experiment 2.

**Section 5B. Experiment 2A- Single-Trial Perceptual Control: Extended Results**

The effect of decision criteria instruction showed the same general pattern observed in Experiments 1 and 2, IB<PoE<BaRD


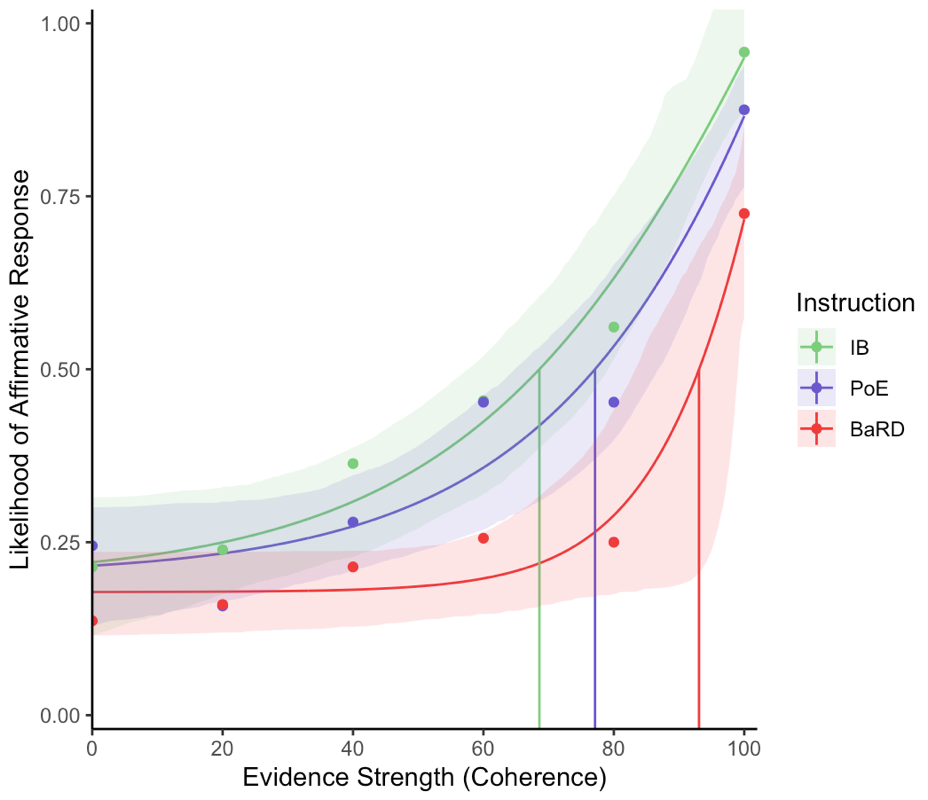


**Fig. S5.1.** Likelihood of an affirmative response by evidence strength (% coherence) and instruction type for single trial dot motion experiment. Shaded regions are 95% confidence intervals estimated via 1000 bootstrap samples. Decision thresholds are marked with vertical lines.

**Section 6A. Experiment 3- Decision Costs: Extended Methods**

**Participants.** We recruited 3688 participants from the United States via Amazon Mechanical Turk (44% male, 56% female, 24 non-binary; Mean age=38.00 years, range=18-89). We recruited participants until we reached roughly 40 observations per cell after all exclusions, as in Experiment 1. Participants were paid $0.75 for successfully completing the survey. The time to completion was between five and eight minutes on average. All participants provided informed consent, and the experimental protocol was approved by the Vanderbilt University Institutional Review Board.

**Task Design.** The task employed a 4 (domain: legal, medical, scientific, control; within-subjects) x 9 (objective evidence strength: 0%, 20%, 40%, 60%, 80%, 90%, 95%, 99%, 100%; within subjects) x 3 (decision criteria instruction: no instruction or “intuitive belief”-IB, PoE, BaRD; within-subjects) x 2 (decision cost: low, high; within-subjects) design. Participants responded to four scenarios in random order, one scenario from each domain, with random assignment to scenario, objective evidence strength, decision criteria instruction, and cost level. Thus, like the experiments above, each participant only saw a single legal scenario. After the four experimental scenarios, participants completed an attention check scenario that was identical to the trial scenarios in its design but contained specific language instructing participants to provide a specific response on the next screen (Fig. S6.1).

Participants completed the study on the Qualtrics online survey platform at their own pace. The procedure was the same as explained in Expt. 1 except as indicated below. After participants read the instructions and clicked to continue (or immediately after the scenario for trials in the IB condition), two outcome-related sentences appeared on the screen (Fig. S6.1). The first sentence stated the outcome if the participant gave an affirmative response (e.g. “If you believe that Mark stole the company’s data, he will be required to pay a $10,000 fine”); this sentence always included the cost to the person(s) in the scenario, which was manipulated as low or high (see below). The second sentence stated the outcome if the participant did not give an affirmative response, which was always that nothing would occur (e.g. “If you do not believe that Mark stole the company data, no action will be taken against Mark”). These two sentences were followed by the decision prompt (e.g. “Do you believe that Mark stole the company’s data?”, “Do you believe that Mark will develop Huntington’s disease?”). Participants read the potential decision outcomes and decision prompt and made a yes/no response. On a new page they then provided their own subjective probability for the scenario.

**Scenarios.** One scenario set was from the legal domain. The other (non-legal) scenario sets were designed to assess by comparison specific aspects of the legal scenarios. Given our hypothesis that decisions may be more conservative in the legal domain because participants infer a punishment cost to the individual defendant, the control scenarios were constructed to assess the effect of decisions cost in domains other than legal and to determine whether such costs mattered if they were to an individual or to a community. Specifically, in addition to the Individual Legal scenario set, the experiment included the following sets: Individual Medical- associated with the cost of medical treatment to an individual; Individual General- associated with costs to an individual outside of a legal or medical context; and Community General- associated with costs to a community outside of a legal or medical context. The non-legal results presented in the main text are collapsed across all of these domains to follow the same reporting scheme used in the first experiment, but in the supplementary material we break down the results per domain (see Supplementary Section 7B).

Each scenario was associated with a decision outcome cost for an affirmative response and for a negative response. The outcome for a negative response was always that no action would occur (i.e. no cost). By contrast, the cost of the outcome for an affirmative response had two levels, low or high, which were selected so as to be realistic with respect to the scenario content, with the high cost level always roughly 10x the cost of the low cost level (e.g. fine of $10,000 vs $100,000). (We note that the designation of a cost as low versus high was only meaningful relative to one another within any given scenario. In other words, the ‘low’ cost of one scenario – say $10,000 – may actually be high for another scenario, as the costs within a scenario had to be realistic and we presented a wide range of cost values across scenarios). We included both monetary costs and costs that represented a loss to the individual in duration terms (e.g. duration of incarceration, length of physical incapacitation following treatment), with at least one monetary and one duration cost in each domain. The monetary costs ranged between $5,000, $10,000, $50,000, $100,000, and $1,000,000. Importantly, some of these monetary costs (i.e. $10,000 and $100,000 costs) were common across scenario domains, thus allowing for a direct comparison between domains for the same cost. The duration costs ranged between 1 Day, 10 Days, 1 Month, 3 Months, 6 Months, 1 Year, 1.5 Years, 5 Years, 10 Years, and 100 Years. Similar to the monetary costs, some of the duration costs (i.e. 1 Day and 10 Days) were common across the individual general and community general domains as long as it was realistically possible to use similar duration costs across different scenarios. All scenarios and their possible outcomes are included in Supplementary Section 6C.

**Individual Legal.** These scenarios consisted of three fact patterns (stealing company data, stealing prescription drugs, and murder) crossed with three types of evidence (video facial recognition, finger prints, and DNA). The objective evidence strength is the level of certainty with which investigators concluded that the evidence was left by the protagonist, Mark, presented as a frequentist measure of probability with random assignment to one of nine levels within-subject: 0%, 20%, 40%, 60%, 80%, 90%, 95%, 99%, 100%. The cost levels (low vs high) for each fact pattern were as follows: stealing company data- $10,000 versus $100,000 fine; stealing prescription drugs- 6 months versus 5 years in prison; murder- 10 versus 100 years in prison.

**Individual Medical.** These scenarios consisted of three fact patterns related to the medical test result and potential diagnosis for a patient, Mark. Participants judged the likelihood of either: the patient developing Huntington’s disease based on DNA markers, the patient having Irritable Bowel Disease (IBD) based on a stool sample, or a patient having optic nerve damage based on their intraocular pressure. The objective evidence strength was again presented as a frequentist probability (same levels as above) for the level of certainty of Mark having the disease given the test result. The cost levels (low vs high) for each fact pattern were as follows: Huntington’s disease- $10,000 versus $100,000 per year out-of-pocket treatment; Irritable Bowel Disease (IBD)- 1 month versus 1 year liquid diet following treatment; optic nerve damage- $5,000 versus $50,000 per year out-of-pocket treatment.

**Individual General.** These scenarios consisted of three fact patterns describing the likelihood (i.e. objective evidence strength as frequentist probability) of either: Mark’s small business needing to purchase specific liability insurance within the next year based on a risk management assessment, an incoming electronic packet needed for Mark’s work containing a virus based on detection software analysis, or Mark’s house remaining on the market for 6 months based on diagnostic features of the house. The cost levels (low vs high) for each fact pattern were as follows: Insurance: $10,000 versus $100,000 per year additional liability insurance; Virus: 1 day versus 10 days unable to access the packet for work; Real Estate: $10,000 versus $100,000 decrease in the asking price of the house.

**Community General.** These scenarios consisted of three fact patterns describing the likelihood (i.e. objective evidence strength as frequentist probability) of either: above average temperatures developing in the Pacific Ocean (which would threaten local fish populations) based on meteorological patterns, a lake being unsafe for swimming based on the sulfate content, or interstellar debris damaging a telecommunications satellite based on astronomical measurements. The cost levels for each fact pattern were as follows: Water Temperature- 3 months versus 1.5 years restricted access and fishing for local coastal region; Lake Water- $100,000 versus $1,000,000 cost to lake residents to resorb sulfates; Interstellar Debris- 1 Day versus 10 Days widespread US mobile phone disruption due to moving the satellite out of the path of the debris.

**Statistical Analyses.** Analytical procedures were identical to those used in Experiment 1.


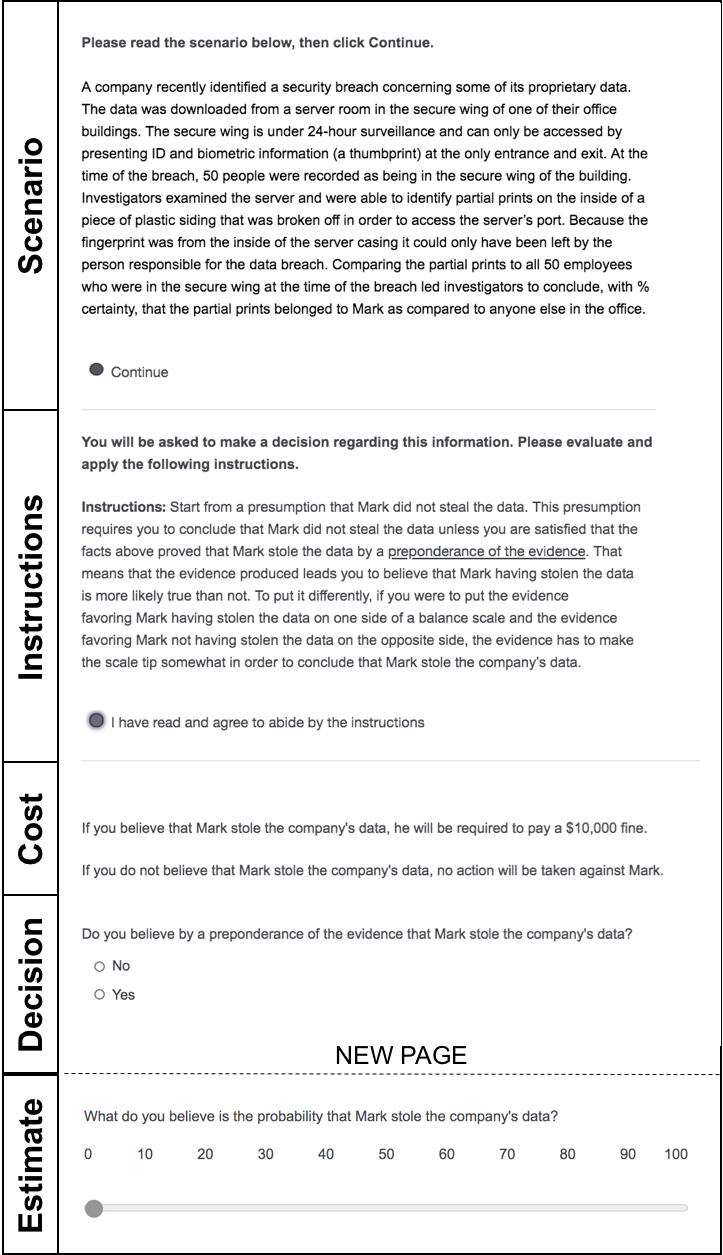


**Fig. S6.1.** Sample trial for Experiment 3 as seen by participants in the legal domain (company data theft x fingerprint evidence scenario) with PoE instruction and low level cost.

**Section 6B. Experiment 3- Decision Costs: Extended Results**


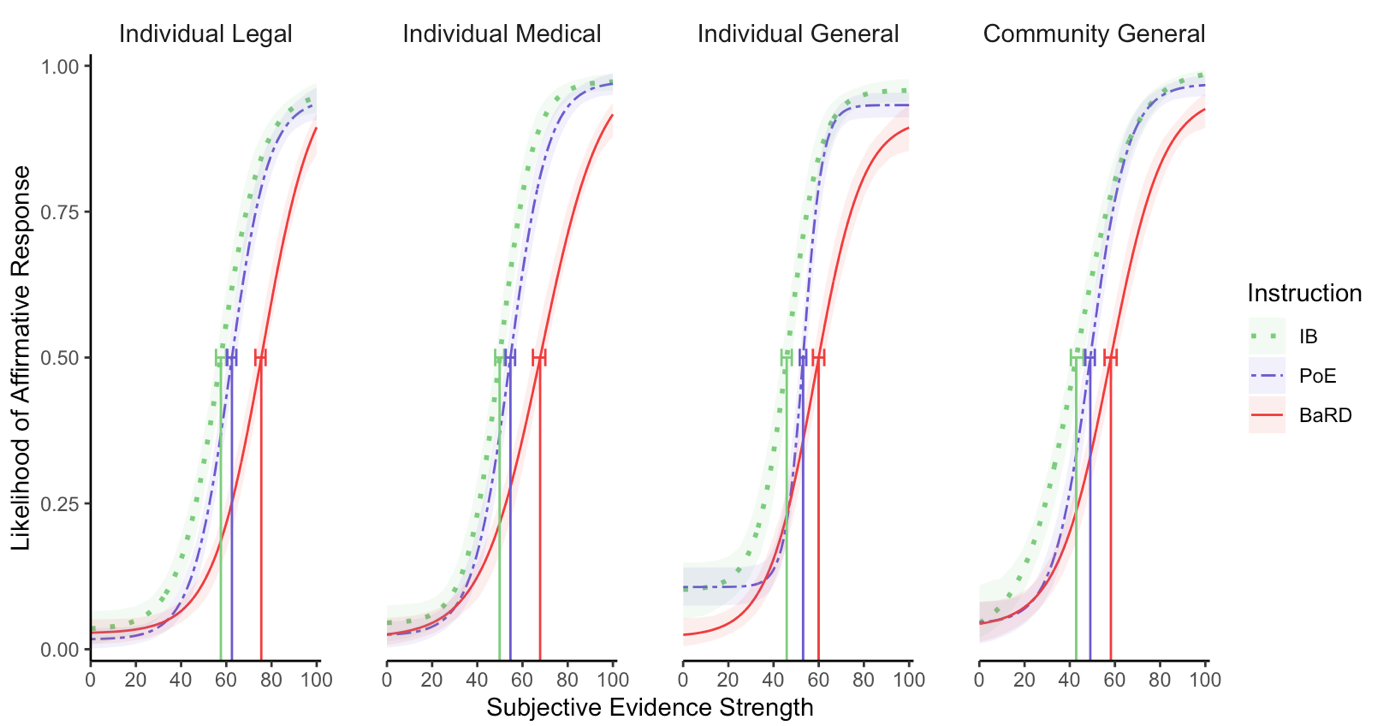


**Fig. S6.2.** Likelihood of an affirmative response by subjective evidence strength and instruction within domain. Shaded regions are 95% confidence intervals estimated via 1000 bootstrap samples. Decision thresholds are marked with vertical lines and 95% error bars.


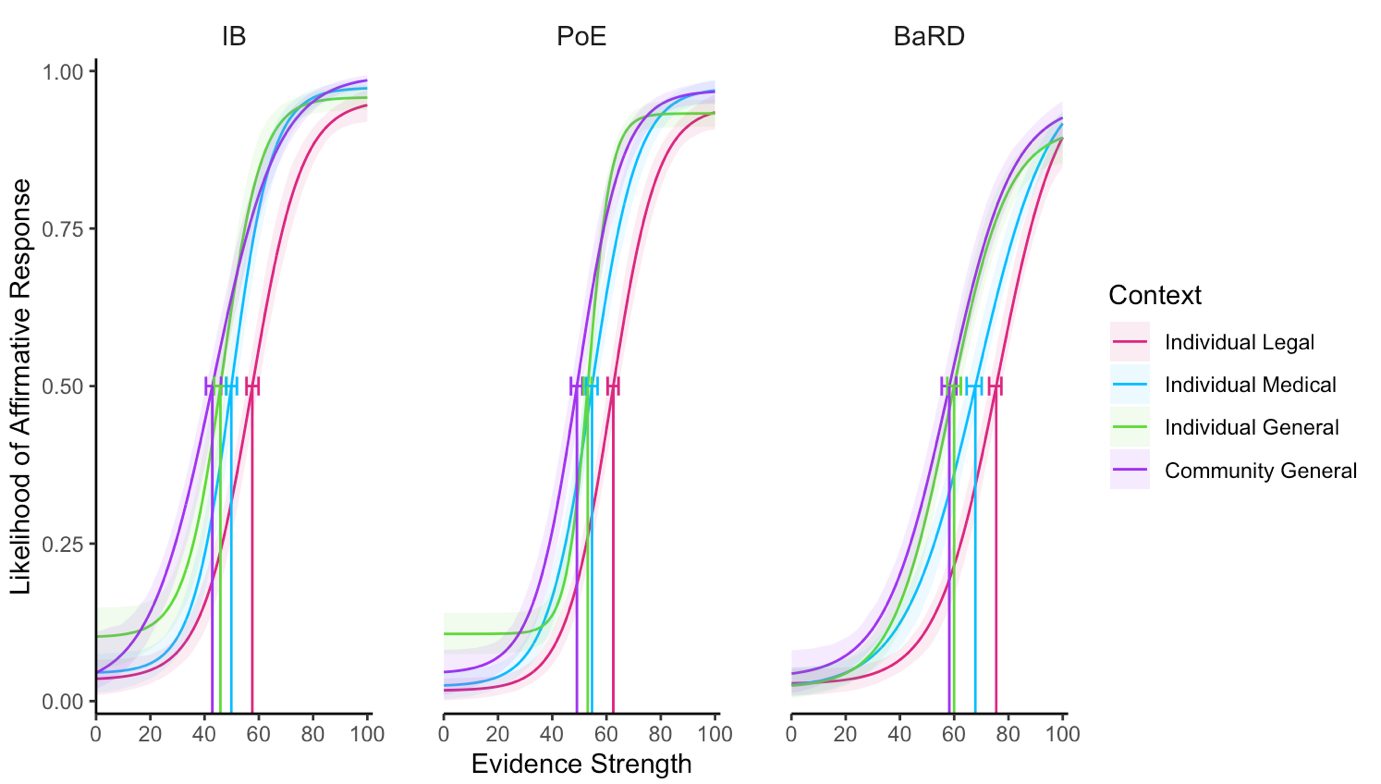


**Fig. S6.3.** Likelihood of an affirmative response by subjective evidence strength and instruction within domain. Shaded regions are 95% confidence intervals estimated via 1000 bootstrap samples. Decision thresholds are marked with vertical lines and 95% error bars.

**Table S6.1A.** Pairwise differences and Bonferroni-corrected CIs for instruction x domain. Confidence intervals that do not contain 0 indicate a significant difference (indicated by *). Control refers to General domain.

**
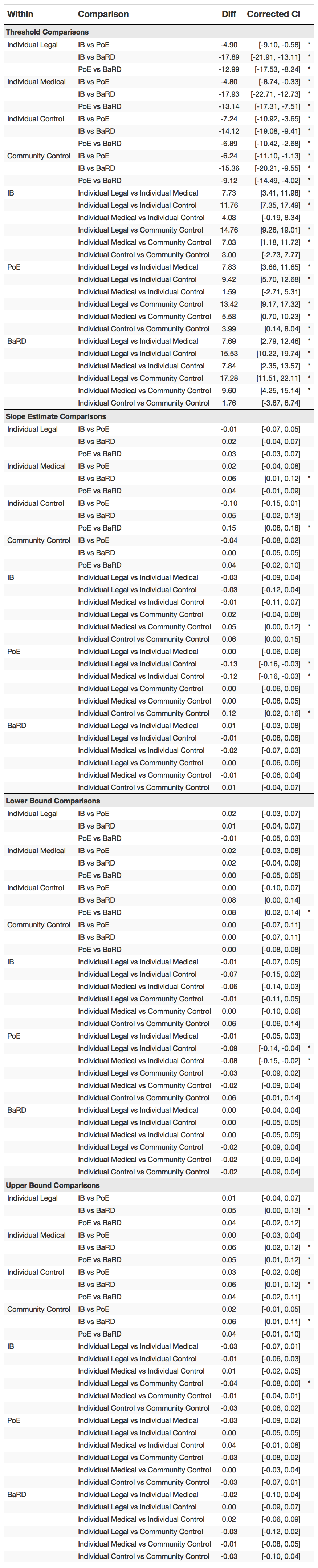
**

**Table S6.1B.** Pairwise differences and Bonferroni-corrected CIs for instruction x domain. Confidence intervals that do not contain 0 indicate a significant difference (indicated by *).

**
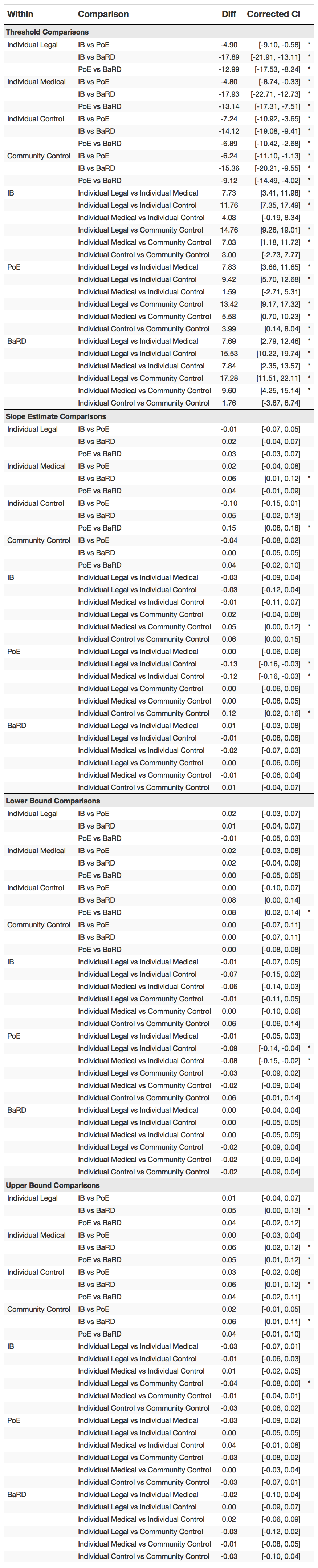
**


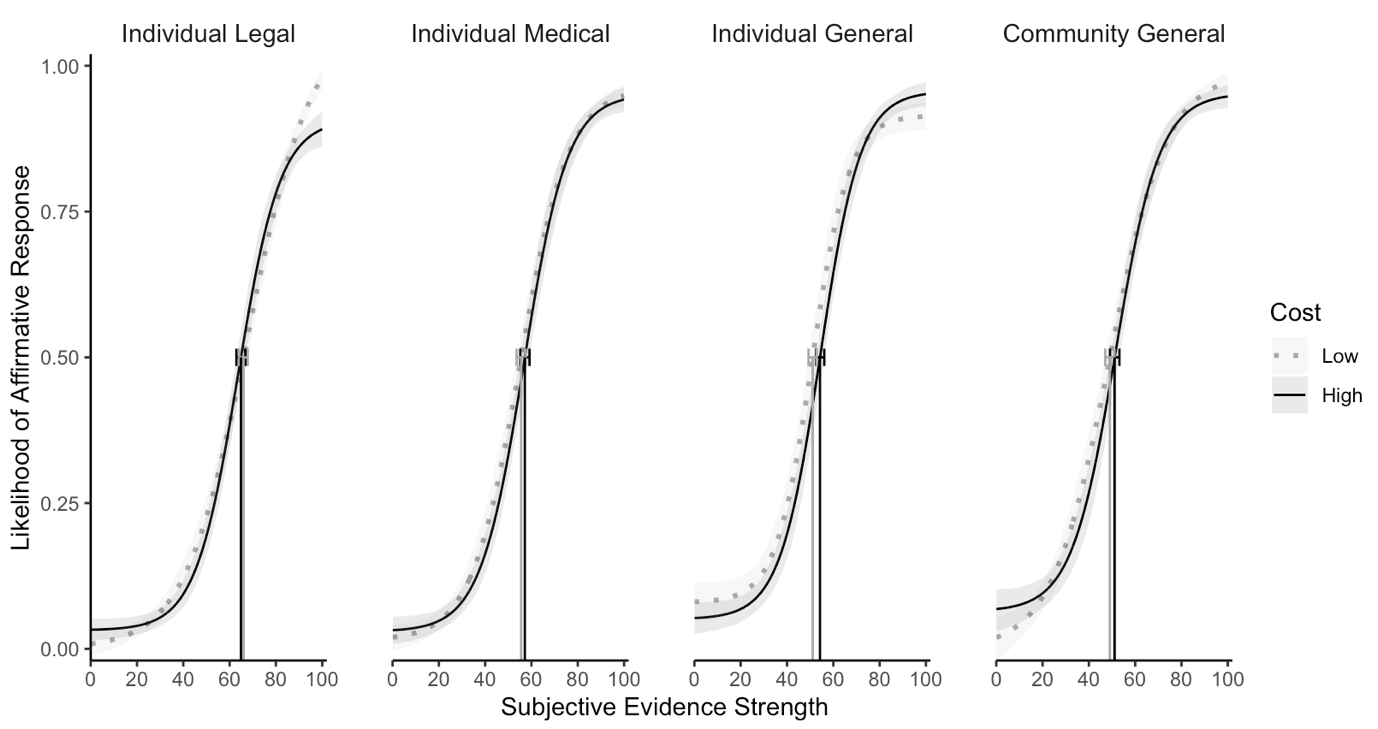


**Fig. S6.4.** Likelihood of an affirmative response by subjective evidence strength and relative cost level within domain. Shaded regions are 95% confidence intervals estimated via 1000 bootstrap samples. Decision thresholds are marked with vertical lines and 95% error bars.


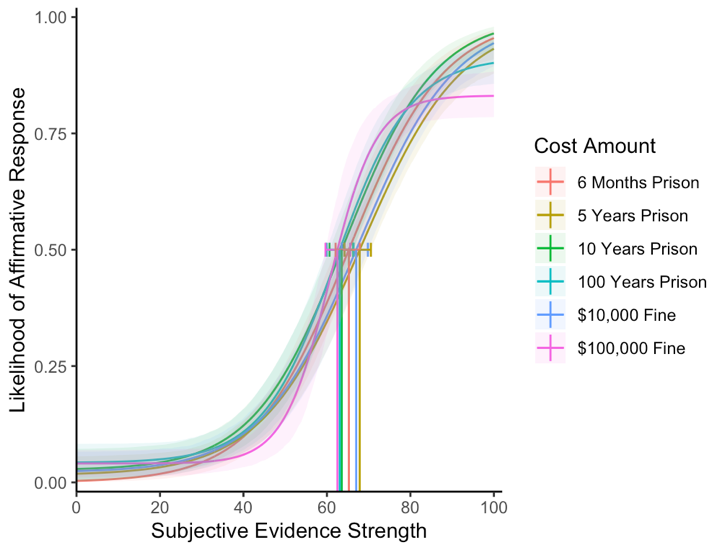


**Fig. S6.5.** Likelihood of an affirmative response by subjective evidence strength and cost amount for legal scenarios. Shaded regions are 95% confidence intervals estimated via 1000 bootstrap samples. Decision thresholds are marked with vertical lines and 95% error bars.

**Table S6.2.** Pairwise differences and Bonferroni-corrected CIs between legal scenario cost amounts. Confidence intervals that do not contain 0 indicate a significant difference (indicated by *).


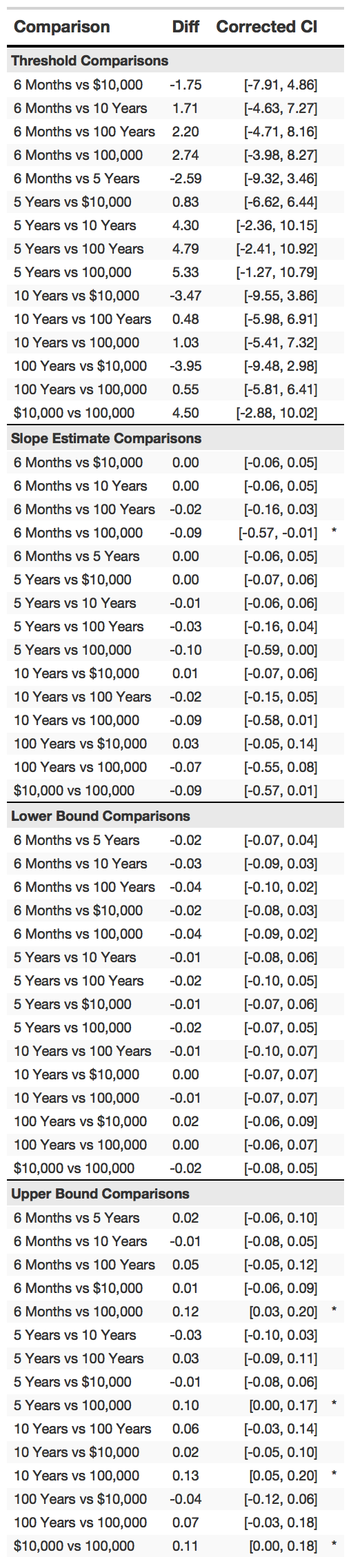

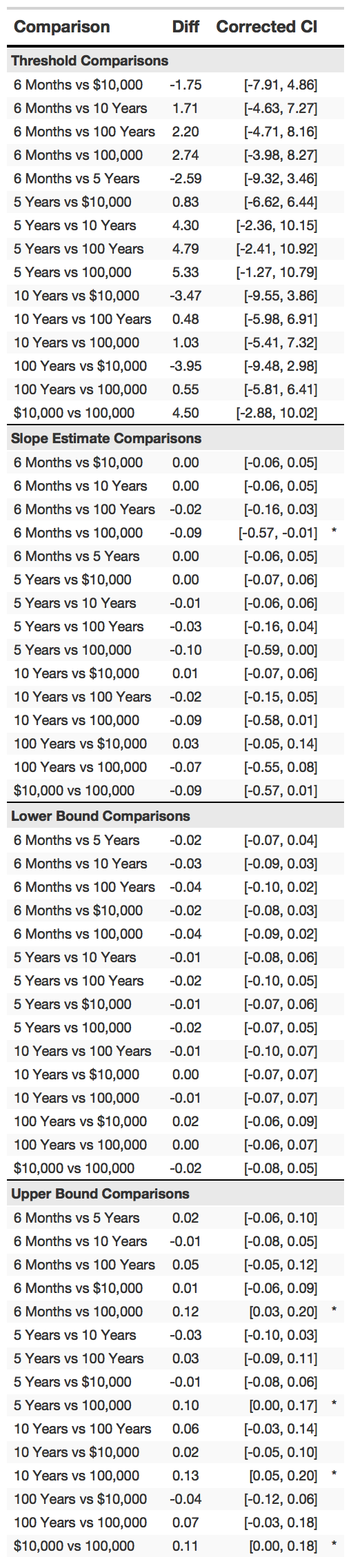


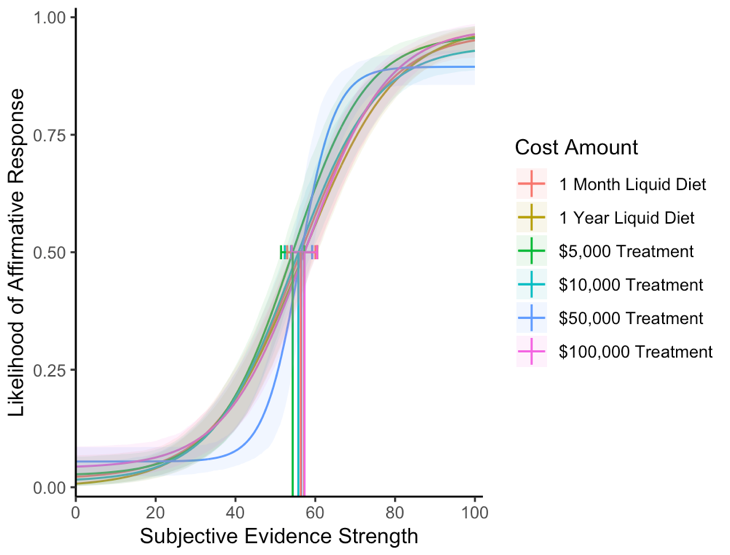


**Fig. S6.6.** Likelihood of an affirmative response by subjective evidence strength and cost amount for medical scenarios. Shaded regions are 95% confidence intervals estimated via 1000 bootstrap samples. Decision thresholds are marked with vertical lines and 95% error bars.

**Table S6.3.** Pairwise differences and Bonferroni-corrected CIs between medical scenario cost amounts. Confidence intervals that do not contain 0 indicate a significant difference (indicated by *).


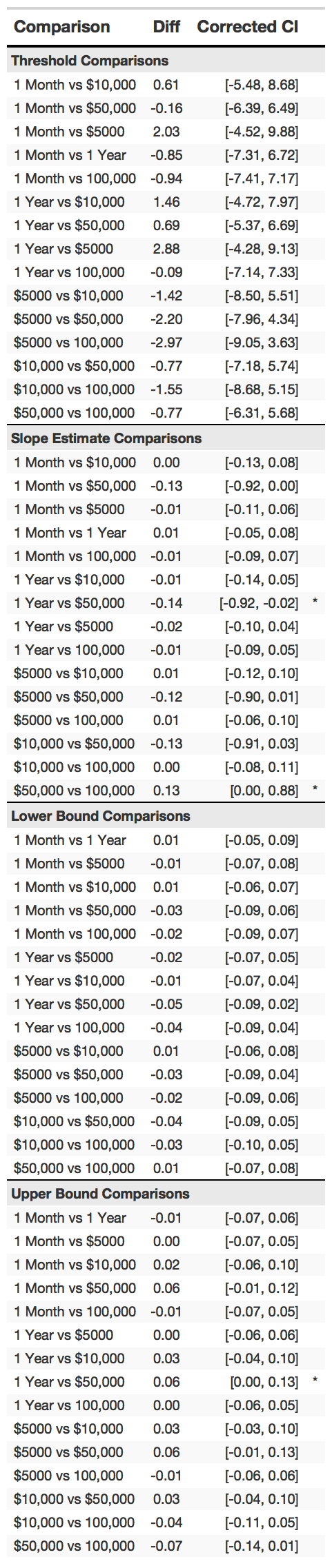

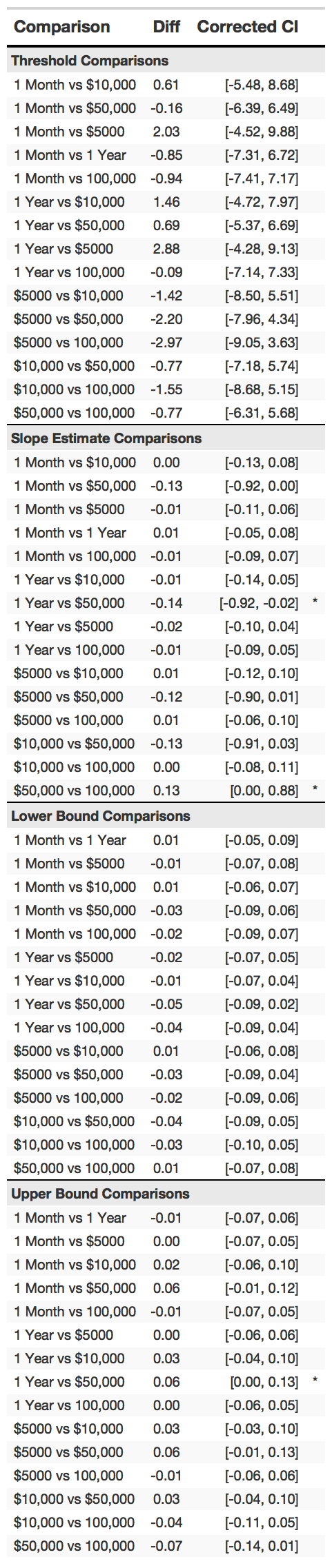


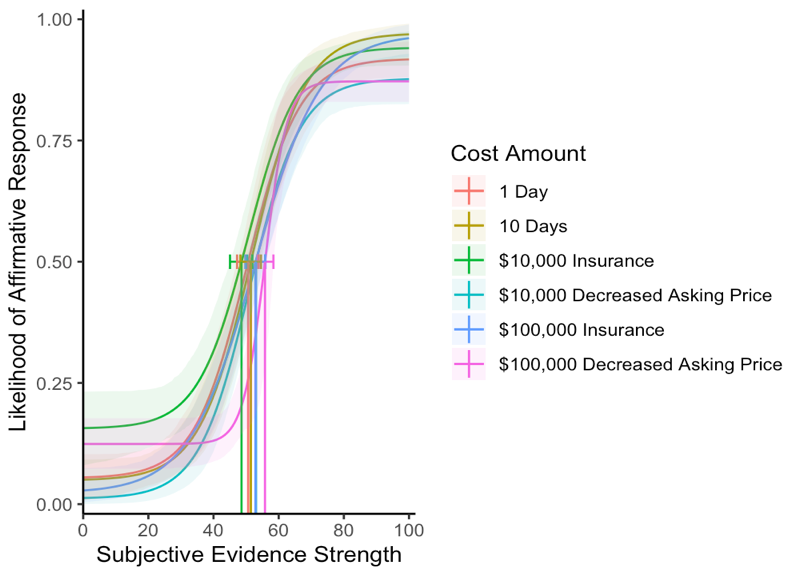


**Fig. S6.7.** Likelihood of an affirmative response by subjective evidence strength and cost amount for individual general scenarios. Shaded regions are 95% confidence intervals estimated via 1000 bootstrap samples. Decision thresholds are marked with vertical lines and 95% error bars.

**Table S6.4A.** Pairwise differences and Bonferroni-corrected CIs between individual general scenario cost amounts. Confidence intervals that do not contain 0 indicate a significant difference (indicated by *)


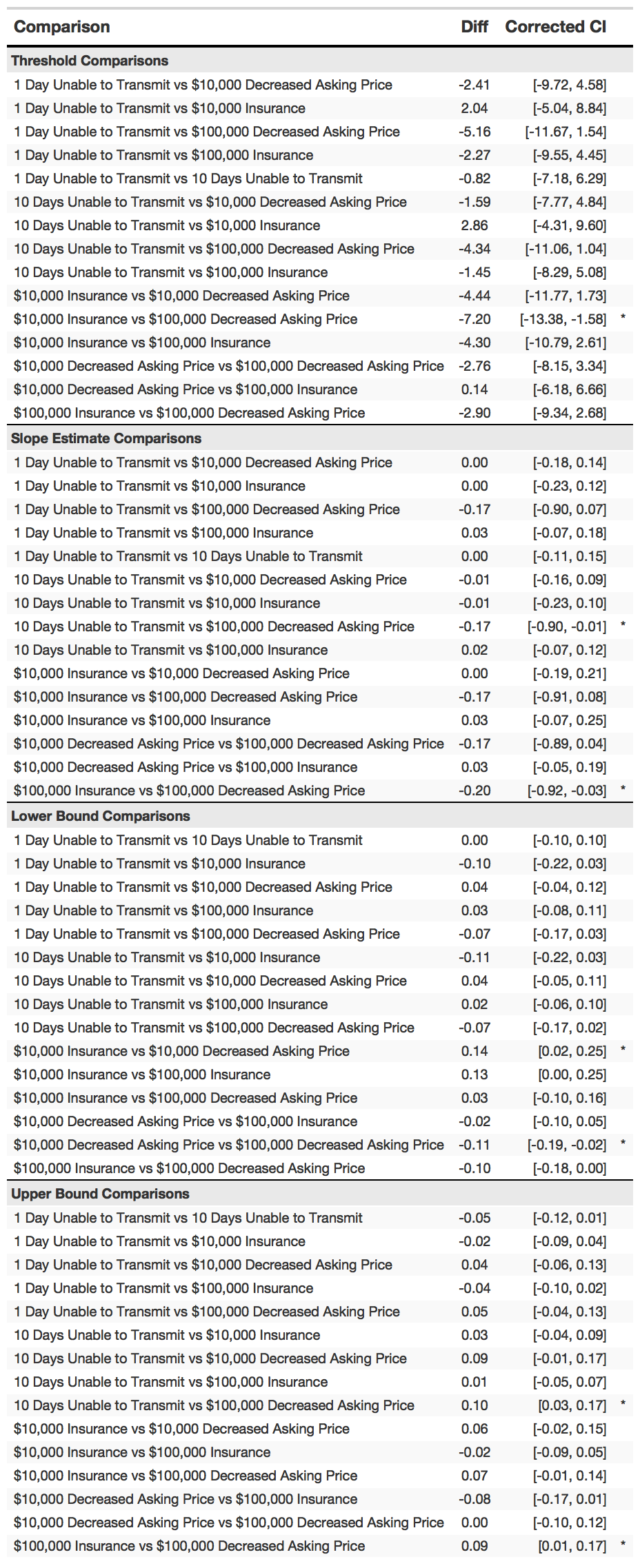


**Table S6.4B.** Pairwise differences and Bonferroni-corrected CIs between individual general scenario cost amounts. Confidence intervals that do not contain 0 indicate a significant difference (indicated by *).


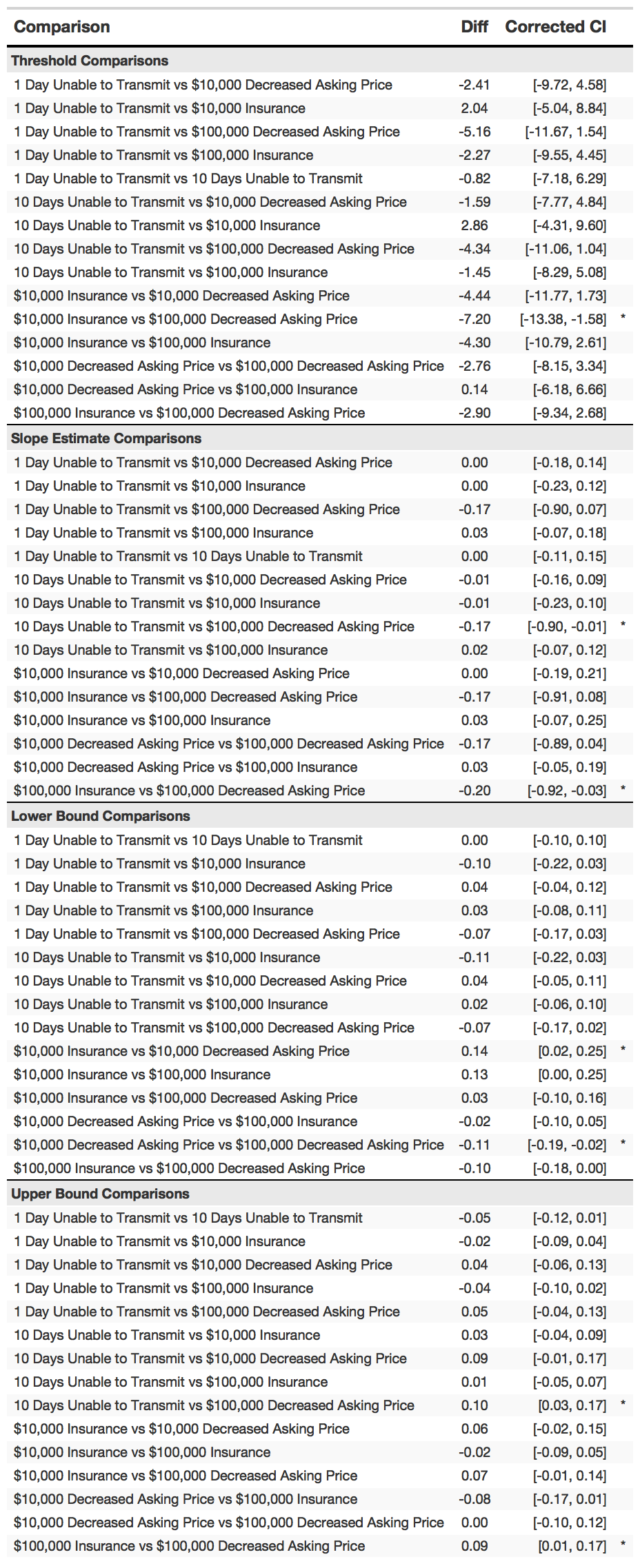


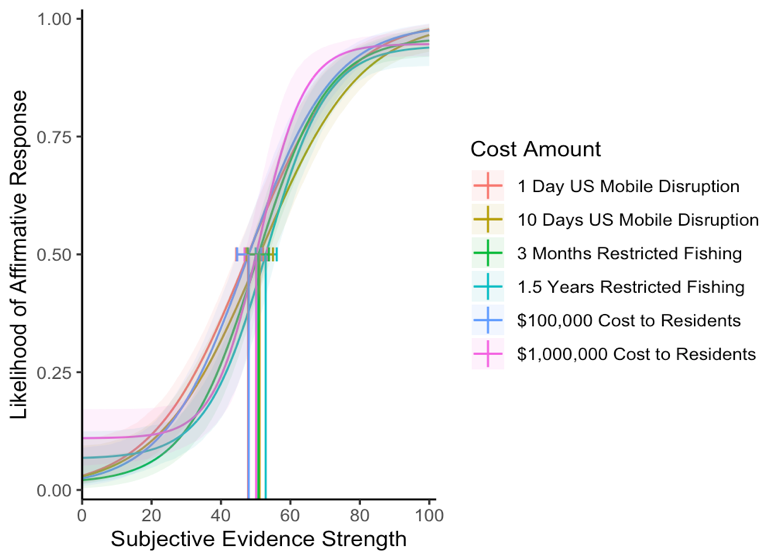


**Fig. S6.8.** Likelihood of an affirmative response by subjective evidence strength and cost amount for community general scenarios. Shaded regions are 95% confidence intervals estimated via 1000 bootstrap samples. Decision thresholds are marked with vertical lines and 95% error bars.

**Table S6.5.** Pairwise differences and Bonferroni-corrected CIs between community general scenario cost amounts. Confidence intervals that do not contain 0 indicate a significant difference (indicated by *).


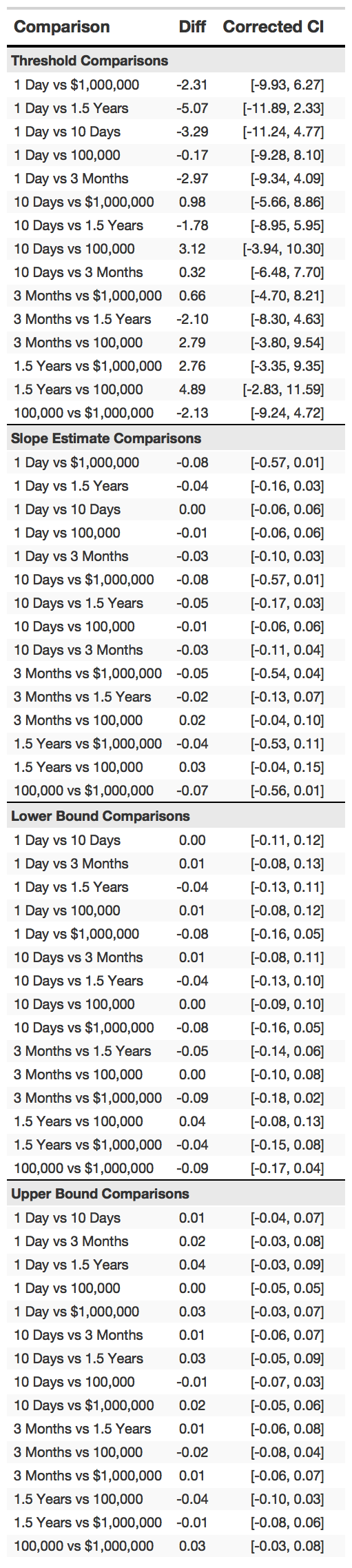

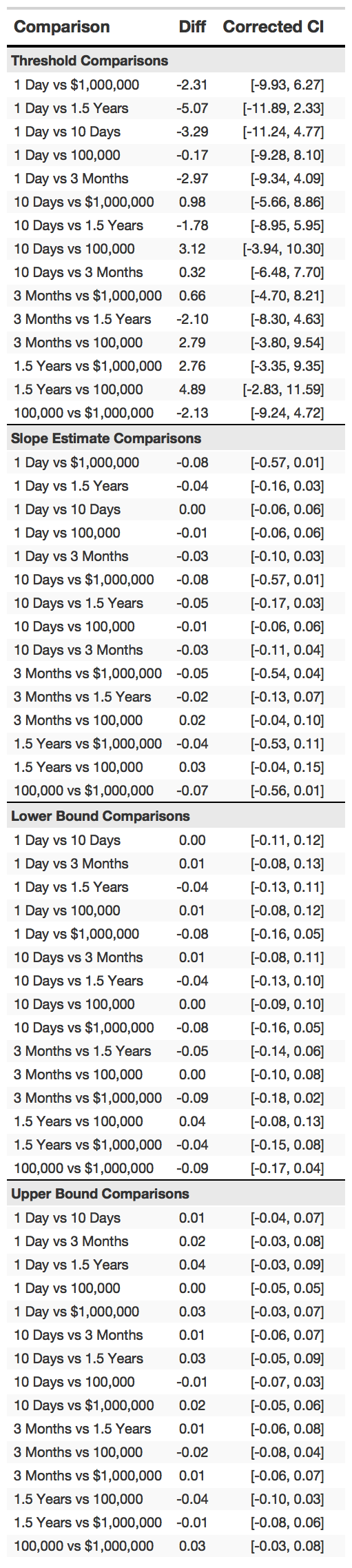


**Table S6.6.** Pairwise differences and Bonferroni-corrected CIs between Experiment 1 legal context (No Cost) and Experiment 3 legal context (Low and High Cost). Confidence intervals that do not contain 0 indicate a significant difference (indicated by *).


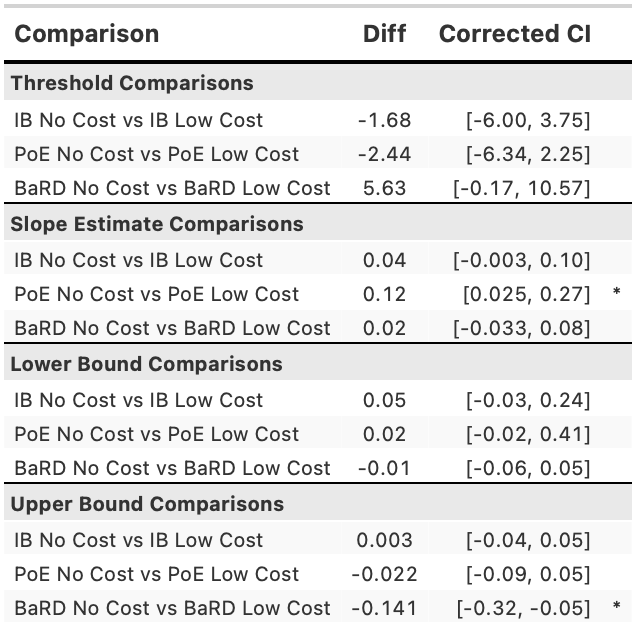


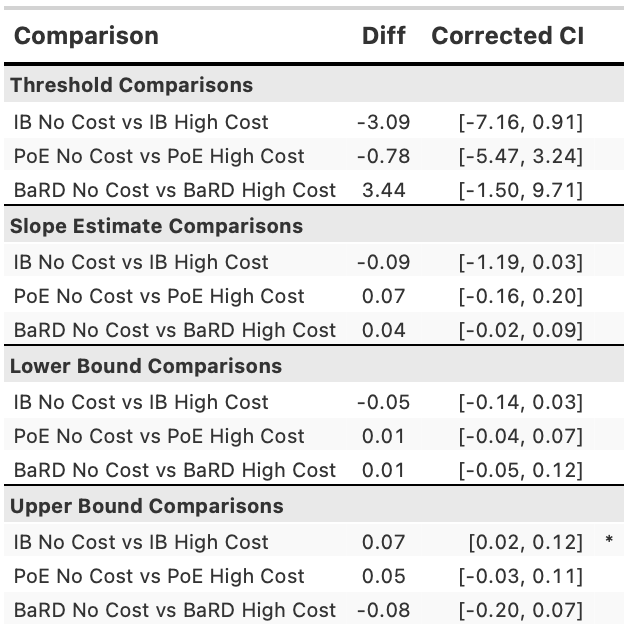


**Table S6.7.** Pairwise differences and Bonferroni-corrected CIs between the thresholds for the $10,000 cost amounts in Experiment 3. The legal context is the $10,000 Fine. Confidence intervals that do not contain 0 indicate a significant difference (indicated by *).


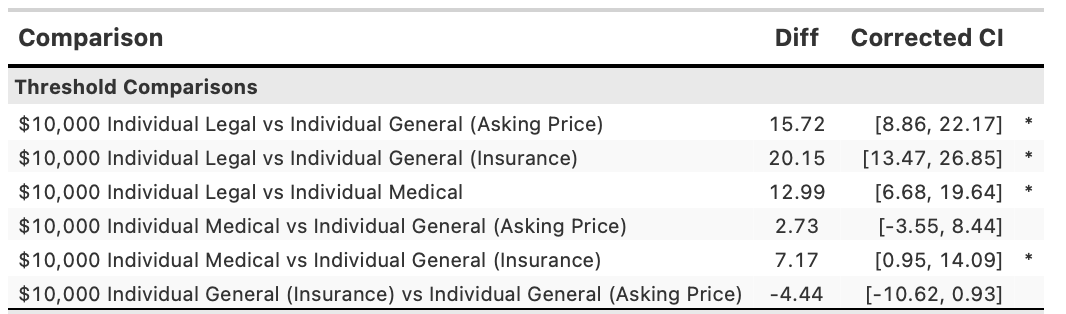


**Table S6.8.** Pairwise differences and Bonferroni-corrected CIs between the thresholds for the $100,000 cost amounts in Experiment 3. The legal context is the $100,000 Fine. Confidence intervals that do not contain 0 indicate a significant difference (indicated by *).


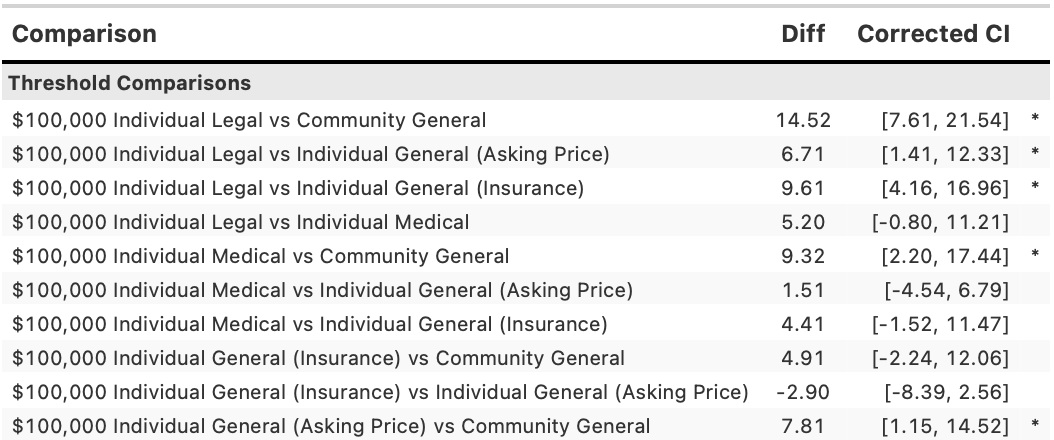


**Section 6C. Experiment 3- Decision Costs: Full Scenarios**

Legal: Stealing company data x Finger prints

A company recently identified a security breach concerning some of its proprietary data. The data was downloaded from a server room in the secure wing of one of their office buildings. The secure wing is under 24-hour surveillance and can only be accessed by presenting ID and biometric information (a thumbprint) at the only entrance and exit. At the time of the breach, 50 people were recorded as being in the secure wing of the building. Investigators examined the server and were able to identify partial prints on the inside of a piece of plastic siding that was broken off in order to access the server’s port. Because the fingerprint was from the inside of the server casing it could only have been left by the person responsible for the data breach. Comparing the partial prints to all 50 employees who were in the secure wing at the time of the breach led investigators to conclude, with [Objective Evidence] % certainty, that the partial prints belonged to Mark as compared to anyone else in the office.

**Low**: If you believe that Mark stole the company’s data, he will be required to pay a $10,000 fine.

OR

**High:** If you believe that Mark stole the company’s data, he will be required to pay a $100,000 fine.

If you do not believe that Mark stole the company’s data, no action will be taken against Mark.

Do you believe that Mark stole the company’s data?

Legal: Stealing prescription drugs x Finger prints

A hospital has recently found that a large amount of prescription drugs went missing from its secure inventory area. The drugs were documented and videotaped being delivered from the manufacturer to the hospital, and drug inventory staff are searched before and after entering the secure area, leaving investigators puzzled. After hearing of a similar incident at another hospital across the country, investigators checked the trash bins in the secure inventory area over the course of a month and found an unmarked envelope that contained hundreds of the missing pills, removed from the container they arrived in. Investigators examined the pills and envelope and were able to identify partial prints on them. Comparing the partial prints to all 50 people who had access to the secure inventory area led investigators to conclude, with [Objective Evidence] % certainty, that the partial prints belonged to Mark as compared to any other employee.

**Low:** If you believe that Mark stole the prescription drugs, he will be sentenced to serve 6 months in prison.

OR

**High:** If you believe that Mark stole the prescription drugs, he will be sentenced to serve 5 years in prison.

If you do not believe that Mark stole the prescription drugs, no action will be taken against Mark.

Do you believe that Mark stole the prescription drugs?

Legal: Murder x Finger prints

While at sea a young female crewmember of the tanker was found raped and murdered after having been missing for 3 days. There were no eyewitness accounts or security cameras, and there was not enough physical evidence to be of use. The knife used to commit the murder was found with the body, covered with the victim’s blood. Investigators examined the knife and were able to identify partial prints in the dried blood on the handle, which were the only fingerprints left on the entire knife. Comparing the partial prints to all 50 males onboard the ship led investigators to conclude, with [Objective Evidence] % certainty, that the partial print belonged to Mark as compared to anyone else on board.

**Low:** If you believe that Mark murdered the female crewmember, he will be sentenced to serve 10 years in prison.

OR

**High:** If you believe that Mark murdered the female crewmember, he will be sentenced to serve 100 years in prison.

If you do not believe that Mark murdered the female crewmember, no action will be taken against Mark.

Do you believe that Mark murdered the female crewmember?

Non-Legal: Huntington’s disease

A genetic test is performed on Mark by using a small cotton swab to collect a sample of cells from the inside of his cheek. The sample is then securely sent to a laboratory where it is processed by certified technicians to determine the order of the nucleotides in the individual’s genetic code. The nucleotides that make up DNA include adenine (A), thymine (T), cytosine (C), and guanine (G). The results of Mark’s genetic test reveal that a specific section of his DNA contains 36 repeats of the ‘CAG’ nucleotide sequence. When an individual is found to have this many repeats of the ‘CAG’ sequence it can be concluded, with [Objective Evidence] % certainty, that they will go on to develop Huntington’s disease.

**Low:** If you believe that Mark will develop Huntington’s disease, he will receive an experimental treatment that slows Huntington’s progression at a cost to him of $10,000 per year out of pocket.

OR

**High:** If you believe that Mark will develop Huntington’s disease, he will receive an experimental treatment that slows Huntington’s progression at a cost to him of $100,000 per year out of pocket.

If you do not believe that Mark will develop Huntington’s disease, no treatment will be administered to Mark.

Do you believe that Mark will develop Huntington’s disease?

Non-Legal: IBD

A stool sample is collected from Mark. The sample is then securely sent to a laboratory where it is processed by certified technicians in order to determine the amount of fecal calprotectin present. Calprotectin is a protein involved in signaling inflammation in the body and is located predominantly in specific types of white blood cells called neutrophil granulocytes. The results of the Mark’s stool sample reveal a fecal calprotectin level of 400 μg/mg. When an individual is found to have this level of fecal calprotectin, it can be concluded with [Objective Evidence] % certainty that they have Inflammatory Bowel Disease.

**Low:** If you believe that Mark has Inflammatory Bowel Disease, he will undergo a surgical treatment procedure to remove damaged digestive tract tissue, which will put him on a strict liquid diet for 1 month.

OR

**High:** If you believe that Mark has Inflammatory Bowel Disease, he will undergo a surgical treatment procedure to remove damaged digestive tract tissue, which will put him on a strict liquid diet for 1 year.

If you do not believe that Mark has Inflammatory Bowel Disease, no treatment will be administered to Mark.

.

Do you believe that Mark has Inflammatory Bowel Disease?

Non-Legal: Glaucoma

An optometrist performs a tonometry test on Mark by numbing the eye with analgesic eye drops, staining it with dye, and then lightly touching it with a probe. The instrument is used to press gently against the cornea and the cornea resists by pushing back onto the tonometer. This test effectively measures Mark’s intraocular pressure (i.e. the fluid pressure inside the eye). Elevated intraocular pressure can cause damage to the optic nerve. The results of the Mark’s tonometry test reveal a pressure of 22 millimeters of mercury. When an individual is found to have this amount of pressure, it can be concluded with [Objective Evidence] % certainty that they have optic nerve damage.

**Low:** If you believe that Mark has optic nerve damage, he will undergo laser treatments to reduce intraocular pressure at a cost to him of $5,000 per year out of pocket.

OR

**High:** If you believe that Mark has optic nerve damage, he will undergo laser treatments to reduce intraocular pressure at a cost to him of $50,000 per year out of pocket.

If you do not believe that Mark has optic nerve damage, no treatment will be administered to Mark.

.

Do you believe that Mark has optic nerve damage?

Non-Legal: Water temperature

A team of meteorologists is responsible for taking regular, periodic measurements of different weather parameters in the Pacific Ocean, including wind speed and surface temperature. The team gathers these measurements from multiple locations across the ocean at each time point. The meteorologists examine the relationships between the different weather parameters and make predictions about future weather events based on previously observed trends. The meteorologists detected a premature weakening of the Pacific trade winds. When this is observed it can be concluded, with [Objective Evidence] % certainty, that above average temperatures will develop in the Pacific Ocean the following summer, which would threaten local fish populations in a coastal region that is highly dependent on fishing for its economy and food supply.

**Low:** If you believe that above average water temperatures will develop in the Pacific, access to the coastal region will be restricted and fishing will not be permitted for 3 months to preserve fish populations.

OR

**High:** If you believe that above average water temperatures will develop in the Pacific, access to the coastal region will be restricted and fishing will not be permitted for 1.5 years to preserve fish populations.

If you do not believe that above average water temperatures will develop in the Pacific, no action will be taken towards the coastal region.

Do you believe that above average water temperatures will develop in the Pacific Ocean?

Non-Legal: Chemical contamination VERSION B

A company is called to assess the water quality of a small lake. The company can determine the chemical content in water streams and whether any of the present chemical substances are found in enough concentration to indicate water toxicity. The company collects water samples from several locations along the banks of the lake at multiple times to account for the different concentrations of the chemicals that may be in the water. These samples are then rigorously analyzed in a laboratory to determine the contents of the lake water. The company detects sulfates in the water at a level of 10 mg/L. When this level of sulfates is detected it can be concluded, with [Objective Evidence] % certainty, that the lake is unsafe for swimming.

**Low:** If you believe that the lake is unsafe for swimming, the company will introduce precipitates in the lake to resorb the sulfates at a cost of $100,000 to the lake residents.

OR

**High:** If you believe that the lake is unsafe for swimming, the company will introduce precipitates in the lake to resorb the sulfates at a cost of $1,000,000 to the lake residents.

If you do not believe that the lake is unsafe for swimming, nothing will be done to the lake.

Do you believe that the lake is unsafe for swimming?

Non-Legal: Asteroid VERSION B

A division of astronomers oversee and manage the planetary defense operation of NASA. Part of the team is responsible for detecting and tracking near-Earth objects (NEO) and issuing reports for those objects that rise to the level of being potentially hazardous due to a projected impact with Earth or Earth’s satellites. At their last meeting, the astronomers reported an approaching cluster of interstellar debris flying in the direction of a vital telecommunications satellite (as this is a geostationary satellite, it maintains a constant orbit to provide consistent coverage). Given the size and mass of the debris, it can be concluded, with [Objective Evidence] % certainty, that the debris will damage the telecommunications satellite.

**Low:** If you believe that the debris will damage the satellite, the will must be moved out of orbit to avoid the debris, causing widespread mobile phone use disruption in the US for 1 day.

**High:** If you believe that the debris will damage the satellite, the satellite will be moved out of orbit to avoid the debris, causing widespread mobile phone use disruption in the US for 10 days.

If you do not believe that the debris will damage the satellite, the satellite will not be moved.

Do you believe that the debris will damage the telecommunications satellite?

Non-Legal: Virus

Mark is a stockbroker whose income critically relies on time-sensitive stock exchange information transferred over the internet to manage his clients’ financial assets. There is concern about the potential for viruses to infiltrate the network so Mark has implemented a system that detects and prevents viruses using software designed to examine virus presence in the packets that are transmitted on the network. The software examines five factors to determine whether the packet contains a virus. Based on these five factors it can be concluded, with [Objective Evidence] % certainty, that the incoming network packet contains a virus.

**Low:** If you believe that the incoming packet contains a virus, the software will quarantine the packet and make the stock exchange information unavailable for Mark to view or transmit for 1 day.

OR

**High:** If you believe that the incoming packet contains a virus, the software will quarantine the packet and make the stock exchange information unavailable for Mark to view or transmit for 10 days.

If you do not believe that the incoming packet contains a virus, the software will not quarantine the incoming packet and Mark will receive it.

Do you believe that the incoming network packet contains a virus?

Non-Legal: Real Estate

A residential real estate company has worked with computer scientists in their area to develop an algorithm for predicting house sales. The company has identified a myriad of features that contribute to how slow or fast a property moves on the market. These factors include the age of the house, the number of bedrooms, the number of bathrooms, the size of the garage, the presence of a basement, and the height of the ceilings, along with many others. Mark is wanting to sell his house quickly so he can purchase a new one in the city he has relocated to. A real estate agent assesses the features of Mark’s house that is newly on the market for $400,000 and in turn, runs the house through the algorithm. Based on the house’s features, it can be concluded with [Objective Evidence] % certainty that Mark’s house will still be on the market in six months.

**Low:** If you believe that the house will still be on the market in six months, Mark will have to decrease the asking price of the house by $10,000 now to sell sooner.

OR

**High:** If you believe that the house will still be on the market in six months, Mark will have to decrease the asking price of the house by $100,000 now to sell sooner.

If you do not believe that the house will still be on the market in six months, Mark will not adjust the price of his house.

Do you believe that Mark’s house will still be on the market in six months?

Non-Legal: Insurance

Mark owns and operates a small business that uses heavy machinery to produce specialty products for customers. With limited funds (net profits totaled $180,000 last year), he needs to determine whether he should purchase optional specific liability insurance in addition to the base-level liability insurance he has purchased for the business. A risk management assessment tool can predict the need for such specific liability insurance using information about the business, including the type of machinery used, prior business accidents, and number of employees. Mark inputs his business information and uses the risk management assessment tool to determine his insurance needs. Based on the information for Mark’s business it can be concluded, with [Objective Evidence] % certainty, that the business will need to utilize the optional specific liability insurance occur within the next year.

**Low:** If you believe that the business will need to utilize the specific liability insurance, Mark will purchase the optional specific liability insurance at a cost of $10,000 per year.

OR

**High:** If you believe that the business will need to utilize the specific liability insurance, Mark will purchase the optional specific liability insurance at a cost of $100,000 per year.

If you do not believe that the business will need to utilize the specific liability insurance, Mark will not purchase optional specific liability insurance.

Do you believe that Mark’s business will need to utilize the optional specific liability insurance occur within the next year?

Attention Check: Restaurant

A sous-chef at a Michelin Star restaurant is in charge of overseeing the kitchen staff and ensuring the proper preparation of food. As a fine dining establishment, the chefs, cooks, and kitchen hands have to be absolutely vigilant about food safety. To demonstrate that you are reading each scenario, please move the slider to 12 on the next page when asked to indicate a value for what you believe is the probability that the refrigerator was above the approved temperature for 2 hours. This scenario will not be analyzed, you may respond below however you wish. During a busy shift last night, the sous chef noticed that the thermometer on the full refrigerator read 60 degrees Fahrenheit instead of the normal 37 degrees Fahrenheit. When the temperature is this high, it can be concluded with 90% certainty that the refrigerator has been above the approved temperature for 2 hours.

**Low:** If you believe that refrigerator has been above the approved temperature for 2 hours, all of the food in the refrigerator will have to be disposed of at a loss of $5,000 to the restaurant.

OR

**High:** If you believe that the refrigerator has been above the approved temperature for 2 hours, all of the food in the refrigerator will have to be disposed of at a loss of $50,000 to the restaurant.

If you do not believe that the refrigerator has been above the approved temperature for 2 hours, the restaurant will not dispose of any of the food in the refrigerator.

Do you believe that the refrigerator has been above the approved temperature for 2 hours?

**Section 7A. Experiment 3A- Decision Cost Severity Ratings Control: Extended Methods**

In order to determine whether and how individuals differentiate between the costs used in Experiment 3, Experiment 3A had participants rate the severity of the cost associated with each scenario rather than making a binary decision about culpability.

**Participants.** We recruited 165 participants from the US via Amazon Mechanical Turk (53% male, 47% female, mean age=37.64 years, range=19-67), based on a power analysis that indicated roughly 169 participants were needed. Participants who successfully completed the survey received $0.75, with an average completion time between five and eight minutes. All participants provided informed consent, and the experimental protocol was approved by the Vanderbilt University Institutional Review Board.

**Task Design.** The task employed a 4 (domain: legal, medical, scientific, general; between-subjects) x 5 (objective evidence strength: 20%, 40%, 60%, 80%, 100%; within-subjects) x 2 (decision cost: low, high; within-subjects) design. We did not include the instruction manipulation here as we did not find an interaction between instruction and decision outcome costs in Experiment 3. In addition, we only presented a subset of the objective evidence strengths used in Experiment 3 because we were not applying a psychometric approach to analyze this data (we also found no main effect or interaction with objective evidence strength). As in Experiment 3, participants completed four trials, one in each domain, followed by an attention check. Each trial began with the scenario presentation as before. Following the scenario presentation, participants viewed the cost associated with the scenario (similar to cost presentation in Experiment 3; e.g. “If Mark stole the company data, he will be required to pay a $10,000 fine”). We removed the “If you believe” language since participants in this experiment were not asked to render a yes/no decision regarding the scenario. Participants were instead asked “How severe do you believe this potential cost is?” and responded with a mouse by dragging a bar along a scale from 1 (Not at all severe) to 10 (Extremely severe) on the computer screen. The scenarios and costs associated with each scenario were identical to those used in Experiment 3.

**Statistical Analyses.** We conducted a 4 (domain: individual legal, individual medical, individual general, community general) x 2 (cost: low, high) mixed model ANOVA with cost severity rating as the dependent variable.

**Section 7B. Experiment 3A- Decision Cost Severity Ratings Control: Extended Results**

There was a main effect of domain (F(3,652)=10.86, p<.001) and cost (F( 1, 652)=34.57, p<.001), with no interaction between them (F(3, 652)=0.70, p=0.55). High costs were rated as more severe than low costs in all of the domains (Fig. S7.1). Post-hoc pairwise comparisons with a Bonferroni correction for domain found that the mean severity ratings for individual medical and community general were both significantly higher than for individual legal and individual general (p’s<0.003). There was no significant difference between individual medical and community general, or between individual legal and individual general.

**
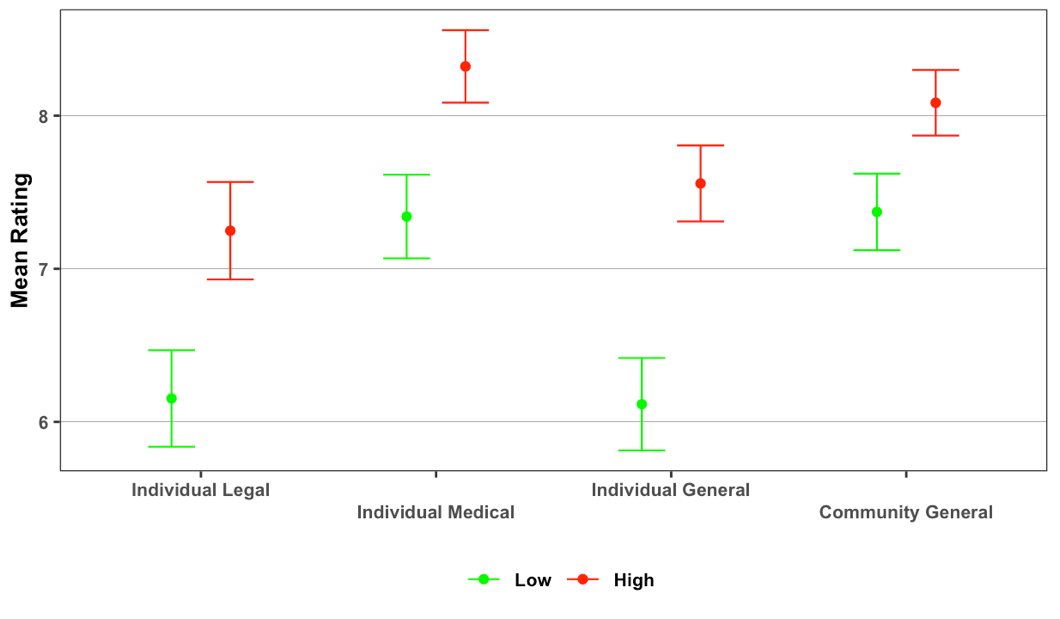
**

**Fig. S7.1.** Mean cost severity rating +/- standard error of the mean, by domain and cost level.

**Supplemental References**

Crump, M. J., McDonnell, J. V., & Gureckis, T. M. (2013). Evaluating Amazon’s

Mechanical Turk as a tool for experimental behavioral research. *PLoS ONE, 8*(3).

Erev, I., Wallsten, T. S., & Budescu, D. V. (1994). Simultaneous over- and underconfidence: The role of error in judgment processes. *Psychological Review, 101*(3), 519-527.

Fründ, I., Haenel, N., & Wichmann, F. (2011). Inference for psychometric functions in the presence of nonstationary behavior. *Journal of Vision, 11*(6), 1-19.

Linares, D. & Lopez-Moliner, J. (2017). quickpsy: An R package to fit psychometric functions for multiple groups. *The R Journal, 8*(1), 122-131.

Gilchrist, J. M., Jerwood, D., & Ismaiel, H. S. (2005). Comparing and unifying slope estimates across psychometric function models. *Perception & Psychophysics, 67*(7), 1289-1303.

Meyniel, F., Schlunegger, D., & Dehaene, S. (2015). The sense of confidence during probabilistic learning: A normative account. *PLoS Computational Biology, 11*(6).

U.S. District Court N. D. Cal, 2012

Wells, G. L. (1992). Naked statistical evidence of liability: Is subjective probability enough? *Journal of Personality and Social Psychology, 62*(5), 739-752.

Wichmann, F. A. & Hill, N. J. (2001a). The psychometric function: I. Fitting, sampling, and goodness of fit. *Perception and Psychophysics*, *63*(8). 1293.

Wichmann, F. A. & Hill, N. J. (2001b). The psychometric function: II. Bootstrap-based confidence intervals and sampling. *Perception and Psychophysics, 63*(8), 1314.
